# Inhibition of AKT1 signaling promotes invasion and metastasis of non-small cell lung cancer cells with K-RAS or EGFR mutations

**DOI:** 10.1038/s41598-017-06128-9

**Published:** 2017-08-01

**Authors:** Guanhua Rao, Mariaelena Pierobon, In-Kyu Kim, Wei-Hsun Hsu, Jianghong Deng, Yong-Wha Moon, Emanuel F. Petricoin, Yu-Wen Zhang, Yisong Wang, Giuseppe Giaccone

**Affiliations:** 1 Lombardi Comprehensive Cancer Center, Georgetown University, Washington, DC USA; 2 Center for Applied Proteomics and Molecular Medicine, George Mason University, Manassas, VA USA

## Abstract

Accumulating evidence supports a role of the PI3K-AKT pathway in the regulation of cell motility, invasion and metastasis. AKT activation is known to promote metastasis, however under certain circumstances, it also shows an inhibitory activity on metastatic processes, and the cause of such conflicting results is largely unclear. Here we found that AKT1 is an important regulator of metastasis and down-regulation of its activity is associated with increased metastatic potential of A549 cells. Inhibition of AKT1 enhanced migration and invasion in KRAS- or EGFR-mutant non-small cell lung cancer (NSCLC) cells. The allosteric AKT inhibitor MK-2206 promoted metastasis of KRAS-mutated A549 cells *in vivo*. We next identified that the phosphorylation of Myristoylated alanine-rich C-kinase substrate (MARCKS) and LAMC2 protein level were increased with AKT1 inhibition, and MARCKS or LAMC2 knockdown abrogated migration and invasion induced by AKT1 inhibition. This study unravels an anti-metastatic role of AKT1 in the NSCLC cells with KRAS or EGFR mutations, and establishes an AKT1-MARCKS-LAMC2 feedback loop in this regulation.

## Introduction

Lung cancer is the leading cause of cancer death^1, 2^ and non-small cell lung cancer (NSCLC) accounts for 80–85% of the cases. Surgery is the mainstay treatment for NSCLC at early stages; however most patients are diagnosed at late stages or recur after surgery, and eventually die of metastatic disease^3^. A better understanding of the molecular mechanisms responsible for NSCLC metastasis is crucial for optimizing the treatment and potentially developing new drugs or strategies against the metastatic process.

Cancer cells frequently acquire genetic and epigenetic alterations that lead to activation of oncogenic signaling pathways, and promote tumor cell growth, survival, migration and invasion^4^. Phosphatidylinositol 3-kinase (PI3K)-protein kinase B (PKB/AKT) is one of the major pathways involved in all these processes, and its inappropriate activation is frequently observed in NSCLC. Hyperactivation of PI3K-AKT signaling can be due to activation of receptor tyrosine kinases (RTKs) or alteration in the specific components within the pathway such as PIK3CA (PI3K catalytic subunit alpha) mutation or deletion of the tumor suppressor phosphatase and tensin homolog (PTEN)^5, 6^. Alteration in this pathway is also known to be one of the mechanisms causal for drug resistance to EGFR inhibitors, for instance, PIK3CA E545K mutation or loss of PTEN^7, 8^.

Although numerous studies implicate a crucial role of PI3K-AKT pathway in the regulation of cell motility, the role of AKT in the control of cancer metastasis remains controversial. In mammals, the AKT kinase family includes three members, AKT1, AKT2 and AKT3, which are encoded by three distinct genes. AKT1 and AKT2 are expressed in most tissues, whereas AKT3 is only expressed in a few organs^9^. Recent studies revealed distinct and conflicting roles of individual AKT members in regulating cell migration and invasion in cells of different origin. AKT1 has been found to inhibit cell migration and invasion by degrading the nuclear factor of activated T cells (NFAT) in human breast cancer cell lines^10^. However, a study using mouse embryonic fibroblasts showed that AKT1 promoted migration whereas AKT2 inhibited migration^11^. AKT1 activation has also been shown to promote melanoma metastasis in a mouse model^12^. Very interestingly, a recent report demonstrated that AKT1 can switch from being a promoter to being a blocker of cell migration and metastasis in breast cancer, as a result of ablation of inositol polyphosphate 5-phosphatase PIPP^13^. Together, these results suggested that differences in genetic background might contribute to the paradoxical roles of AKT in cell migration, invasion and metastasis. Given that several AKT inhibitors are currently in clinical development for cancer intervention^14^, in-depth knowledge of AKT function in these pathological processes is crucial. Also, dissecting the controversial roles of AKT under different genetic backgrounds may impact patient selection for clinical studies of AKT inhibitors in the future.

In this study, we report the identification of AKT1 as a key metastatic regulator in NSCLC cells through Reverse-Phase Protein microArray (RPPA) analysis of a brain-metastasis model. We found that AKT1 signaling was gradually inactivated over four rounds of *in vivo* selection of A549 brain metastases. We demonstrated that AKT1 inhibition promoted migration and invasion of NSCLC cells with KRAS or EGFR mutation *in vitro*, and the pan-AKT inhibitor MK-2206 promoted A549 metastasis *in vivo*. Further, we found that AKT1 inhibition induced phosphorylation of myristoylated alanine-rich protein kinase C substrate (MARCKS) and elevation of LAMC2 protein in the KRAS or EGFR mutant NSCLC cell lines, but not in those with wild type KRAS and EGFR. MARCKS or LAMC2 knockdown abrogated the AKT-inhibition-induced cell migration and invasion. Our results provide first-hand evidence that differences in the genetic background influences the role of AKT1 in tumor invasion and metastasis in NSCLC.

## Results

### AKT1 inactivation is associated with metastatic potential of NSCLC

**Figure 1:**
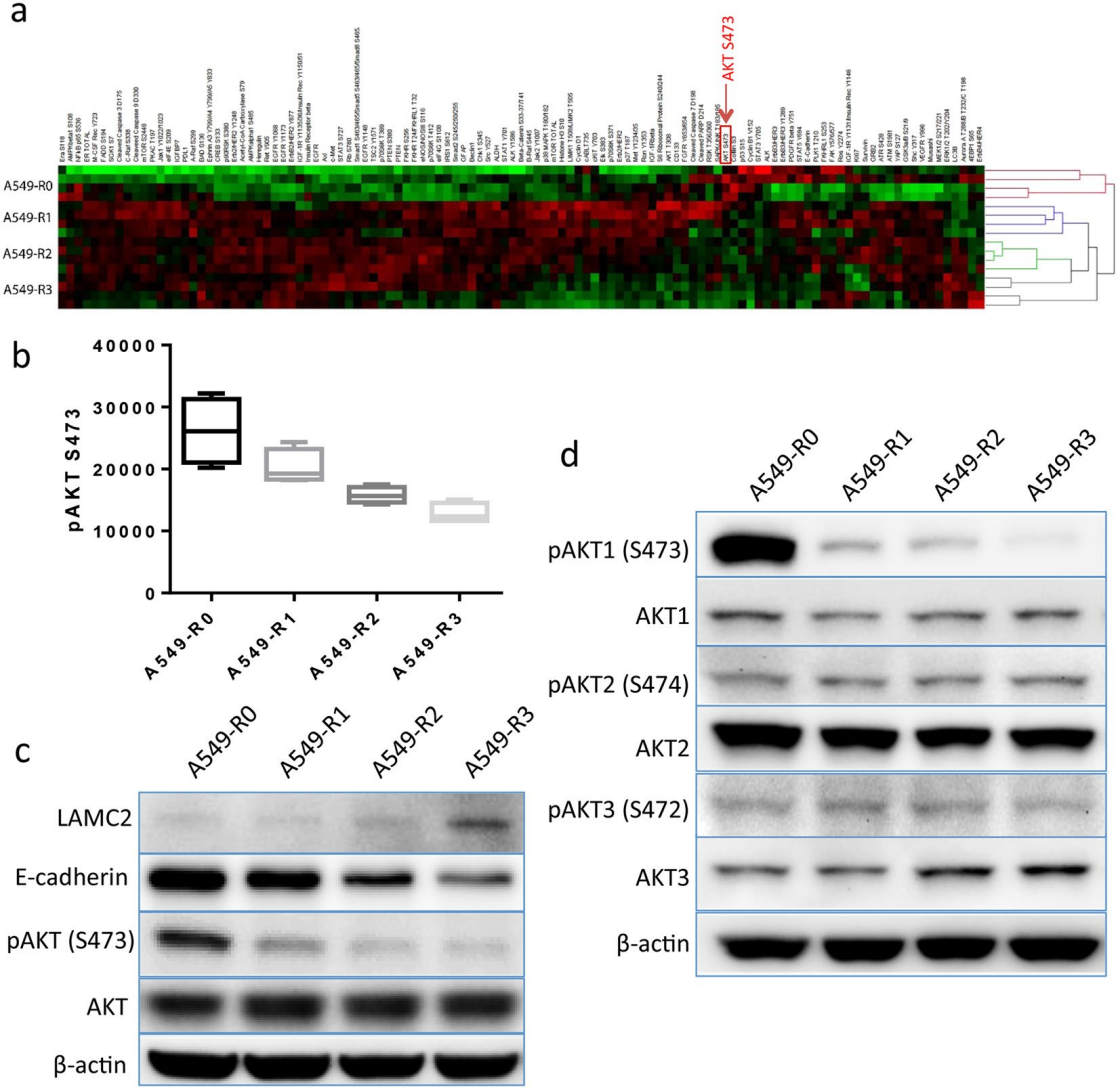
Inactivation of AKT1 signaling correlates with enhanced metastatic potential of A549 cells. (**a**) Heat map of RPPA data derived from A549-R0, -R1, -R2 and -R3 cells. Color scaling of relative protein levels: low (green), median (black) and high (red). (**b**) Signal intensities of S473-phosphorylated AKT quantified from the RPPA data of each subclone. Western blot analyses of (**c**) E-cadherin, pAKT (S473) and total AKT, and (**d**) phosphorylated- and total proteins of AKT1, 2, and 3 in the A549 subclones. ß-actin was used as loading control.

We previously generated four A549 subclones (R1, R2, R3, and R4) from experimental brain metastases through 4 rounds of intracardiac injection of A549 cells or its derivatives into athymic nude mice (Supplementary Fig. S1a and Table S1). The metastatic potential of A549 subclones was significantly increased during this selection, and by round 3, brain metastasis penetrance reached 100%^15^. Changes in protein levels, especially of phosphorylated forms, are known to play critical roles in tumor development and progression, and might not be reflected by genetic and transcriptional alterations^16^. We performed RPPA assay to decipher signaling pathways associated with the enhanced metastatic potential of A549 subclones. This assay includes a panel of antibodies against 114 cancer-associated proteins, including 88 phospho-proteins (Supplementary Table S2)^17^, and we screened for proteins that were differentially expressed in the A549-R0, -R1, -R2 and -R3 cells. Unsupervised clustering of the RPPA data revealed alterations in several canonical signaling pathways (e.g. RTK-MAPK and PI3K-AKT-mTOR) that are known to have roles in the regulation of mitogenesis, cell survival, apoptosis/autophagy, motility/cell adhesion, or inflammatory/immune response (Fig. S1a; Supplementary Fig. S1b and Table S3). Using the Pearson product-moment correlation coefficient analysis, we identified AXL, phosphorylated-Smad1^S463/465^/Smad5^S463/465^/Smad9^S465/467^, phosphorylated-EGFR^Y1068^, total levels of EGFR and phosphorylated-Erb2/HER2^Y1248^ proteins as being significantly increased and strongly correlated with the metastatic potential of A549 subclones (Supplementary Table S3). These results are supported by several other reports^18–20^. Of particular interest, we found that the level of AKT phosphorylation at S473 was inversely correlated with metastatic potential (Fig. 1b and Supplementary Table S3). We further confirmed, by Western blot, that downregulation of pAKT^S473^ in the metastatic subclones was associated with decreased expression of E-cadherin, a marker of epithelial-mesenchymal transition (EMT) (Fig. 1c). We further demonstrated that of the phosphorylated protein expression of the AKT family members, only AKT1 phosphorylation was downregulated in the metastatic subclones (Fig. 1d). These results suggest that inactivation of AKT1 signaling may be correlated with enhanced metastatic potential, implying a potential negative role of AKT1 in the regulation of NSCLC metastasis.

### AKT1 inhibits migration and invasion of cells with KRAS or EGFR mutations

**Figure 2:**
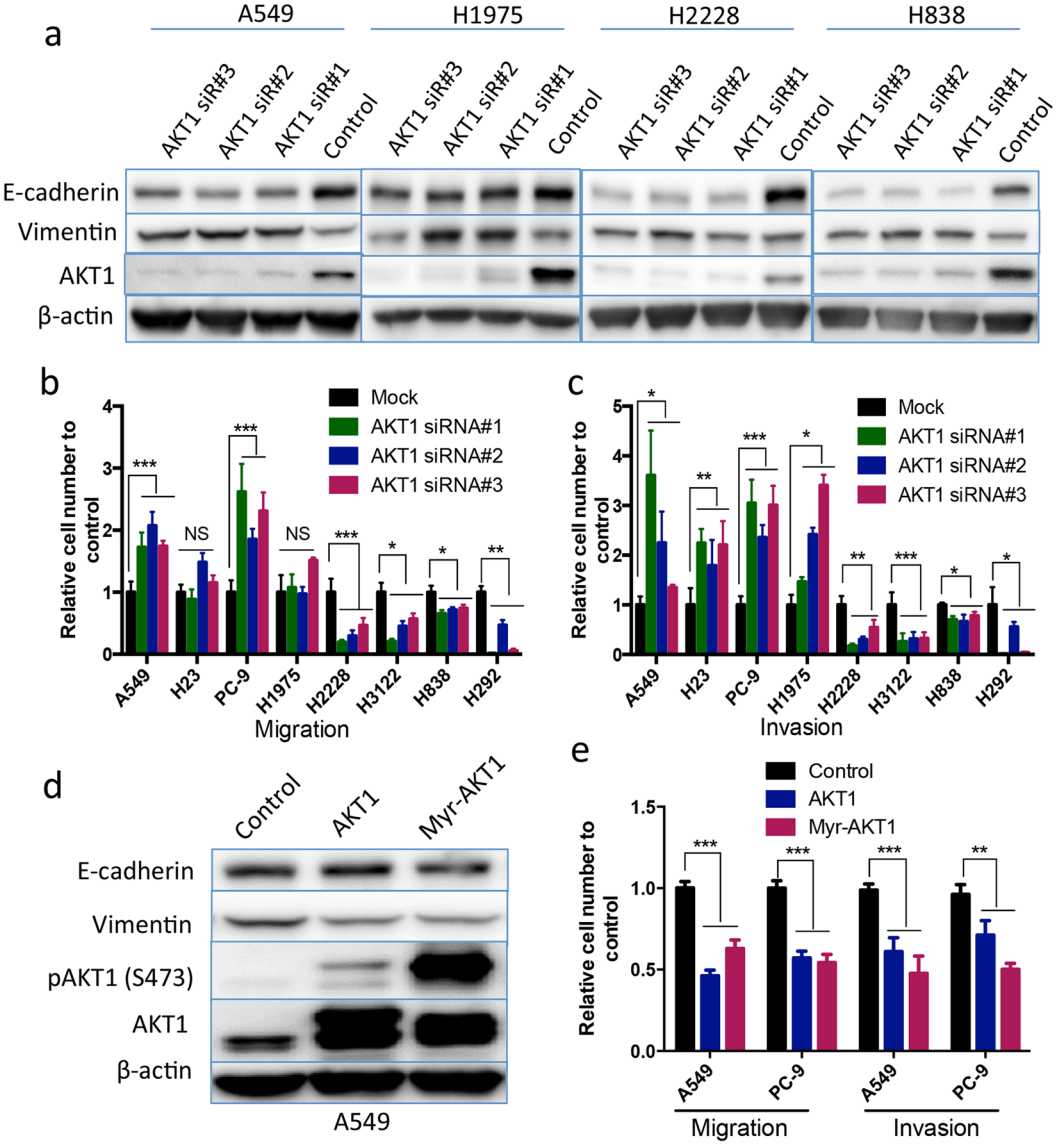
AKT1 inhibits migration and invasion of KRAS or EGFR mutant NSCLC cells. (**a**) Western blot analyses of E-cadherin, Vimentin, and AKT1 in A549, H1975, H2228 and H838 transfected with/without 10 nM of indicated AKT1 siRNA for 48 hours. (**b**) Migration and (**c**) Invasion assays of A549, H23, PC-9, H1975, H2228 H3122, H292 and H838 cells with/without 10 nM of indicated AKT1 siRNA for 48 hours. Graph bar represents mean ± SE. *P < 0.05; **P < 0.01; ***P < 0.001; and NS: not significant. (**d**) Western blot analyses of E-cadherin, Vimentin, phospho- and total AKT1 in A549 cells transfected with/without wild-type AKT1 or a constitutively active form of AKT1 (Myr-AKT1). (**e**) Migration and invasion assays of A549 and PC-9 cells transfected with the indicated expression plasmid for 24 hours. Experiments were carried out in triplicate.

Several studies have demonstrated very different roles of AKT1 in controlling cell migration and metastasis, which varies depending on cell and tissue types^21–26^. To determine whether a difference in genetic background may play a role in such a discrepancy, we examined the function of AKT1 in migration and invasion in a panel of NSCLC cell lines with different driver mutations (Supplementary Table S1). Cell lines include KRAS mutant (A549 and H23), EGFR mutant (PC-9 and H1975), and EML4-ALK translocated cell lines (H2228 and H3122) along with KRAS/EGFR wild type cell lines (H838 and H292). Using three distinct AKT1-specific siRNAs, we found that knockdown of AKT1 reduced the expression of E-cadherin and induced vimentin expression in A549, H1975, H2228 and H838 cells (Fig. 2a). These results suggest that knockdown of AKT1 may induce EMT in these NSCLC cells. Interestingly, AKT1 knockdown significantly increased migration and invasion in A549 and PC-9, invasion only in H23 and H1975 cells, but inhibited migration and invasion in H2228, H3122, H292 and H838 cells (Fig. 2b,c). To evaluate the role of other AKT isoforms, AKT2 and AKT3, in cell migration and invasion, we used AKT isoform-specific siRNA pool to knock down AKT2 and AKT3 in A549, PC-9, H1975 and H838 cells (Supplementary Fig. S2a,b). We found that knockdown of AKT2 had little influence on cell migration and invasion, while knockdown of AKT3 showed a reduction in cell migration and invasion in PC-9, H1975 and H838 cells. These results indicate different roles of AKT isoforms in cancer cell migration and invasion.

Since PI3K-AKT1 signaling plays a crucial role in cell survival, we then asked if the difference in cell migration and invasion was due to a difference of cell survival in these lines as a result of AKT1 knockdown. We knocked down AKT1 in the A549, PC-9, H1975, H2228 and H838 cell lines, and determined cell apoptosis by flow cytometric analysis of Annexin V staining (Supplementary Fig. S3a,b). Knockdown of AKT1 resulted in marginal or no apoptosis, except for H2228 cells in which significant increase of cleaved PARP1 could be detected following AKT1 inhibition (Supplementary Fig. S3c).

The inhibitory role of AKT1 in cell migration and invasion was further explored by exogenous expression of wild-type AKT1 or a constitutively activated form (Myr-AKT1) in A549 and PC-9 cells. We found that overexpression of AKT1 or Myr-AKT1 in A549 cells downregulated vimentin expression, and inhibited migration and invasion of A549 and PC-9 cells (Fig. 2d,e).

Collectively, these results suggest that AKT1 has an inhibitory activity on migration and invasion of NSCLC cells with KRAS or EGFR mutations, underscoring genetic contribution to the apparent paradoxical roles of AKT1 in these cellular processes.

### AKT inhibition by MK-2206 promotes invasion and metastasis in KRAS or EGFR mutant NSCLC models

**Figure 3:**
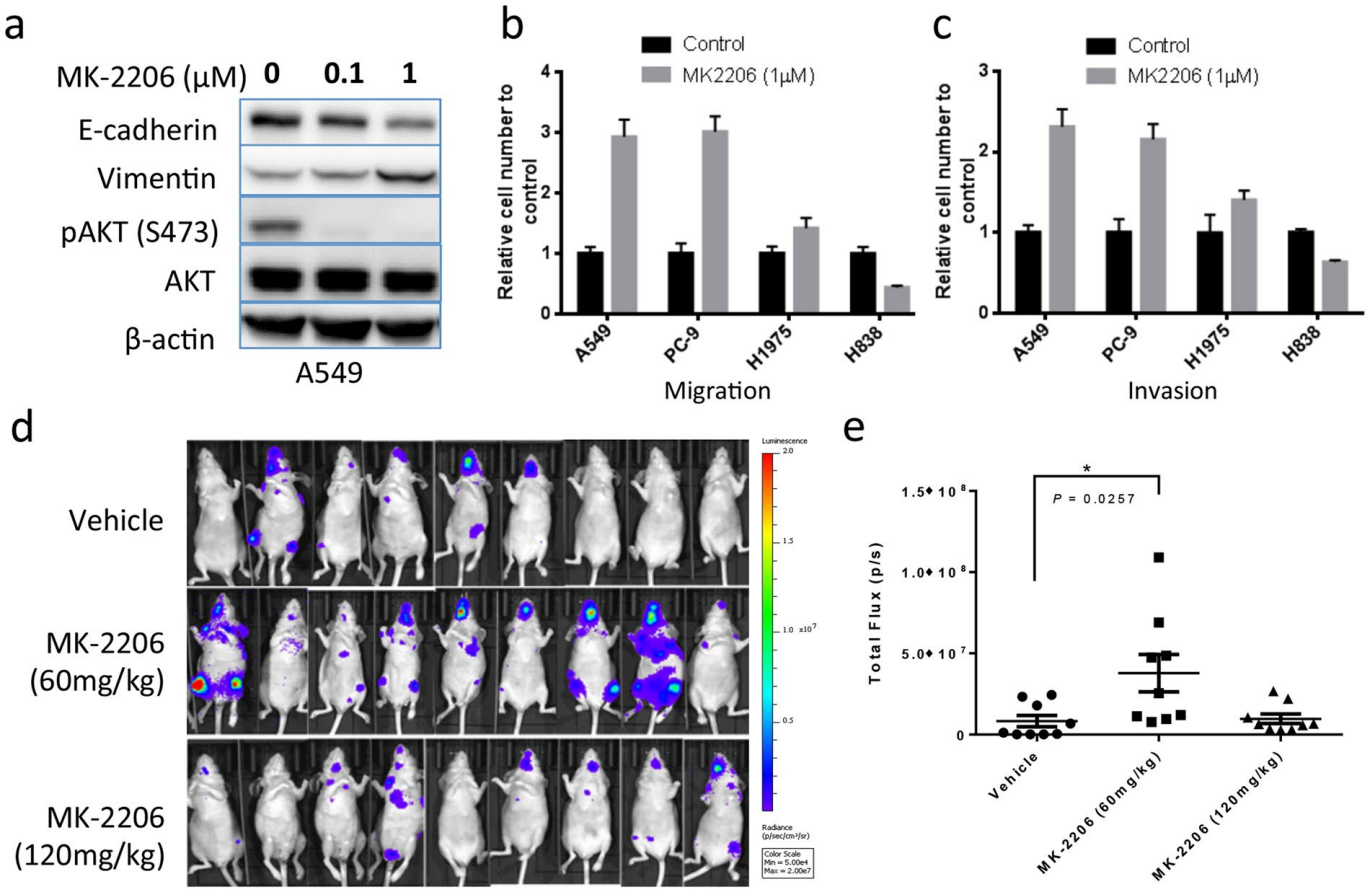
AKT inhibition by MK-2206 promotes invasion and metastasis of NSCLC cells. (**a**) Western blot analyses of E-cadherin, Vimentin, phospho- and total AKT in A549 cells treated with indicated concentration of MK-2206 for 24 hours. (**b**) Migration and (**c**) Invasion of A549, PC-9, H1975 and H838 cells treated with DMSO (Control) or 1 µM MK-2206. (**d**) Experimental metastases of A549 cells in mice treated with vehicle, 60 mg/kg, or 120 mg/kg of MK-2206, visualized by luciferase bioluminescence imaging at week 4 following injection. The imaging parameters were equivalent for all images. (**e**) Quantification of the bioluminescence intensities. Graph bar represents mean ± SE. *Indicates statistic significance (*P* < 0.05).

Given that several agents targeting AKT are in various stages of clinical development^14^, we asked whether AKT inhibitors affect metastatic processes in NSCLC cells, and whether this might impact the therapeutic outcome. We used MK-2206, an allosteric AKT inhibitor presently being investigated in clinical trials, and tested its effect on migration, invasion and metastasis in NSCLC cell lines with and without KRAS or EGFR mutations. Inhibition of AKT by MK-2206 resulted in downregulation of E-cadherin and upregulation of vimentin in A549 cells (Fig. 3a). Further, we found that MK-2206 at 1 µM concentration significantly enhanced migration and invasion of A549, PC-9 and H1975 cells, but inhibited that of H838 cells (Fig. 3b,c). These results are similar to that of AKT1 siRNA knockdown in these cells (Fig. 2), suggesting that the effect of MK-2206 on migration and invasion of these cells is mainly through inhibition of AKT1.

We then asked whether MK-2206 treatment promotes metastasis of KRAS mutant A549 cells using an experimental metastasis model system by intracardiac injection^27^. To enable monitoring *in vivo* metastasis in mice with a bioluminescence imaging system, we stably transfected A549 cells with a luciferase-expressing vector before injection. One day after intracardiac injection of the cells, mice were treated with MK-2206 via oral gavage at 60 mg/kg and 120 mg/kg dosages once daily, three times per week for two weeks, as previously described^28^. Mice in the control group were treated with 30% Captisol, the vehicle used for MK-2206 formulation. Mice were monitored by bioluminescence imaging once weekly. The treatment with 60 mg/kg MK-2206 significantly enhanced A549 metastases, particularly to the brain and bone, based on the intensity of the luciferase reporter activity (Fig. 3d,e). However, no significant difference in the metastasis rates was observed between the groups treated with 120 mg/kg of MK-2206 and with the vehicle. This is likely because high concentration of MK-2206 also causes significant growth inhibition due to its effect on cell viability. *In vitro*, the IC_50_ of MK-2206 in the 3 tested NSCLC cell lines (including A549) ranged from 3.402 µM to 7.929 µM (Supplementary Fig. S4a–c), whereas at 1 µM concentration, it only marginally affected the viability of these cells (Supplementary Fig. S4d). These data indicate that while inhibiting AKT by MK-2206 has anti-tumor activity, it also may promote metastasis of NSCLC cells with certain genetic background.

### Blocking AKT1 induces LAMC2 expression to promote migration and invasion

**Figure 4:**
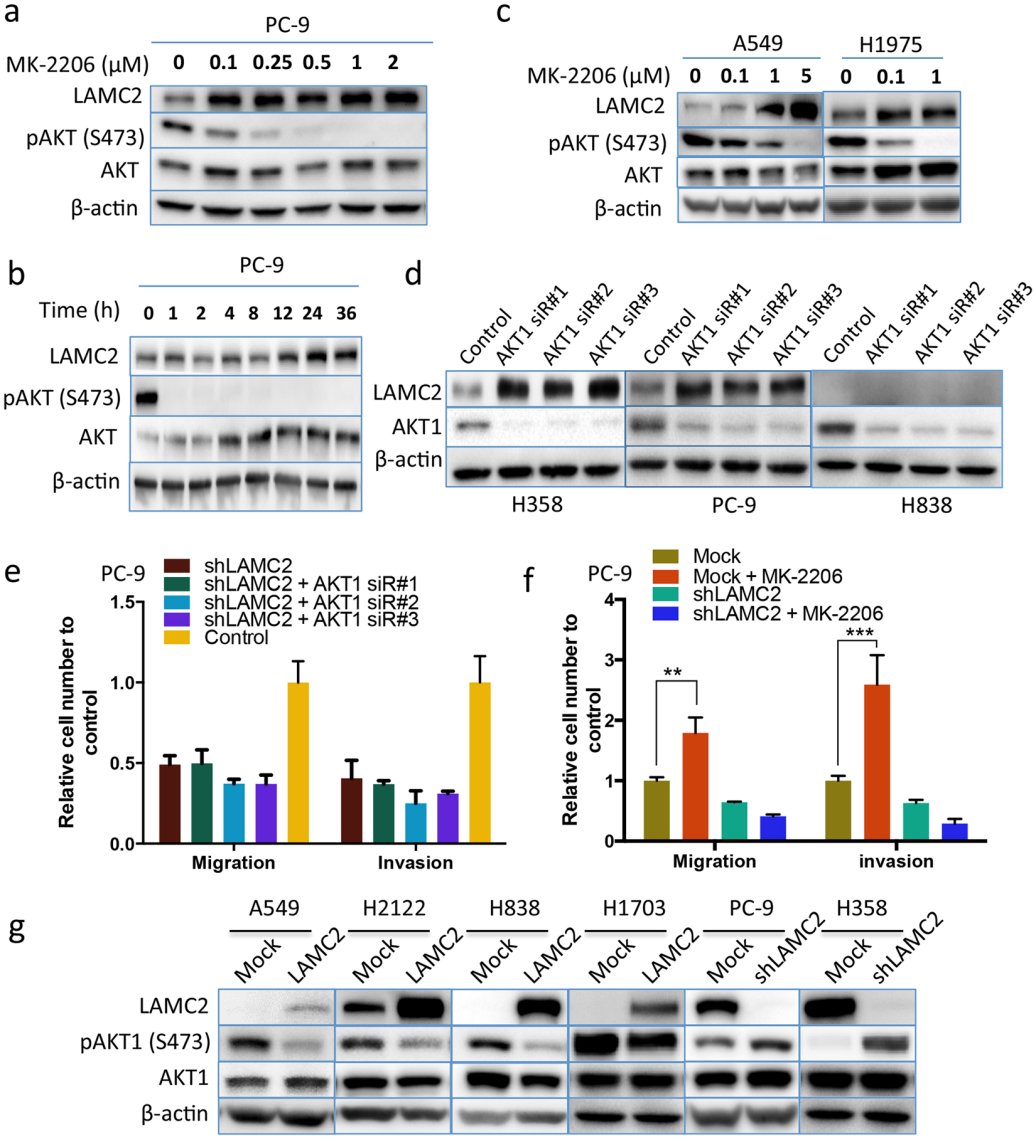
Upregulation of LAMC2 is required for migration and invasion resulting from AKT1 inhibition. Expression of LAMC2 and the phospho- and total AKT in (**a**) PC-9 cells treated with the indicated concentration of MK2206 for 24 hours, (**b**) PC-9 cells treated with 1 µM MK-2206 for the indicated times, and (**c**) A549, and H1975 cells treated with the indicated concentrations of MK-2206 for 24 hours. ß-actin was also detected for loading controls. (**d**) Western blot detection of LAMC2 and AKT1 in H358, PC-9 and H838 cells transfected with three distinct AKT1 siRNAs (10 nM) for 48 hours. Migration and invasion of (**e**) PC-9 cells transfected with LAMC2 shRNA with/without AKT1 siRNA treatment for 48 hours and (**f**) PC-9 cells with/without LAMC2 shRNA and/or 1 μM MK-2206 for 24 hours. ***P < 0.001. (**g**) Western blot analysis of LAMC2 and the phospho- and total proteins of AKT1 in A549, H2122, H838 and H1703 cells stably transfected with LAMC2 expression vector, or in PC-9 and H358 cells infected with shLAMC2 lentiviral expression vector.

LAMC2 was found to promote cell migration and invasion^15, 29^. Previously, we demonstrated that LAMC2 expression is increased in the highly metastatic A549 subclones obtained from experimental brain metastases^15^, and is negatively correlated with AKT1 signaling (Fig. 1b–d). Therefore, we postulated that inhibition of AKT1 promotes cell migration and invasion through upregulation of LAMC2 expression. Indeed, treatment of PC-9 cells with MK-2206 resulted in a significant increase of LAMC2 protein (Fig. 4a) 12 hours after administration and peaked at 24 hours after AKT inhibition (Fig. 4b). Similarly, LAMC2 expression was upregulated in A549 and H1975 cells when treated with MK-2206 for 24 hours (Fig. 4c). To determine the role of AKT1 in this regulation, we examined LAMC2 expression following AKT1 inhibition by siRNA knockdown. Knockdown of AKT1 by three different siRNAs elevated LAMC2 protein in both H358 and PC-9 cells, but not in the KRAS/EGFR wild type H838 cells (Fig. 4d). These data suggest that induction of LAMC2 following AKT1 inhibition is a common event among KRAS or EGFR mutant NSCLC cells.

We then evaluated whether increased LAMC2 impacts on cell migration and invasion induced by AKT1 inhibition. LAMC2 shRNA knockdown inhibited migration and invasion induced by AKT1 siRNA or MK-2206 in PC-9 cells (Fig. 4e and f). Furthermore, LAMC2 knockdown enhanced AKT phosphorylation at S473 in PC-9 and H358 cells, whereas ectopic overexpression of LAMC2 inhibited this phosphorylation in the A549, H2122, H838 and H1703 cells (Fig. 4g). Since mTORC2 (mammalian target of rapamycin complex 2) is recognized as the main kinase phosphorylating AKT at S473^30^, we then investigated whether LAMC2 has any effect on mTORC2. In A549 and H838 cells, high levels of LAMC2 correlated with low protein levels of Rictor, a crucial component of mTORC2, whereas knockdown of LAMC2 promoted Rictor expression in PC-9 cells. However, the phosphorylation levels of mTOR at S2448 were not influenced by LAMC2 level in A549 and H838 cells, and knockdown of LAMC2 could slightly reduce phosphorylation level of mTOR at S2448 (Supplementary Fig. S5). These data suggest the presence of a negative feedback regulation between AKT1 and LAMC2. Upregulation of LAMC2 is required for migration and invasion induced by AKT1 inhibition in the KRAS/EGFR-mutant NSCLC cells.

### MARCKS is a downstream signaling molecule of AKT1 that mediates migration and invasion induced by AKT1 inhibition

Given that induction of LAMC2 by AKT1 inhibition likely required *de novo* synthesis (Fig. 4b), we asked whether this regulation is mediated by the transcription factor FOXO, a downstream target of AKT signaling. FOXO regulates a number of genes involved in cell survival and invasion^31, 32^, and mediates the expression and activation of several receptor tyrosine kinases (RTKs) induced by ATK inhibition, in multiple tumor types^33^. However, collectively knocking down FOXO1, 3 and 4 by a pool of specific siRNAs had almost no effect on LAMC2 expression in A549 and PC-9 cells with or without MK-2206 treatment (Supplementary Fig. S6). These results indicate that induction of LAMC2 by AKT inhibition is not mediated by FOXO.

**Figure 5:**
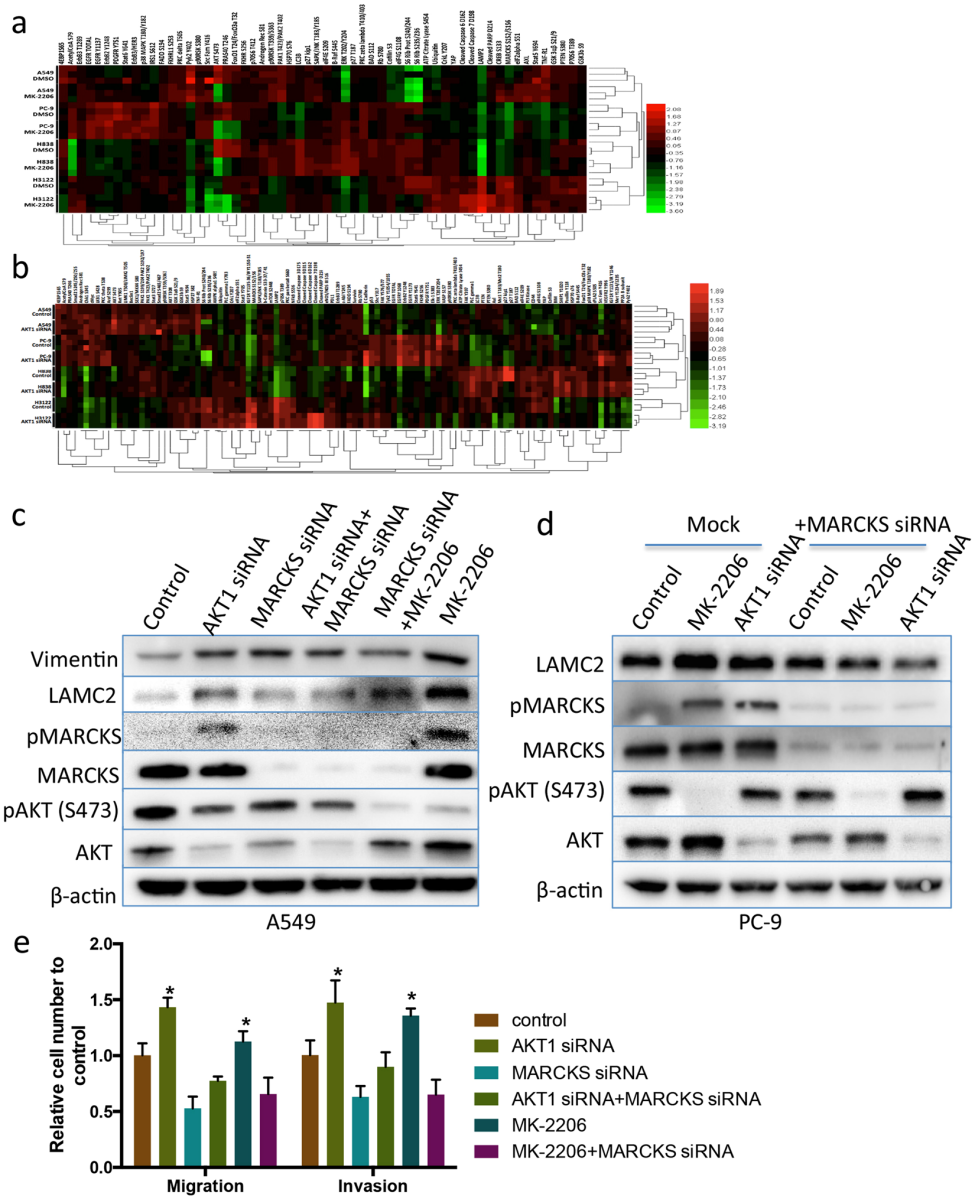
AKT1 inhibition activates MARCKS to promote migration and invasion. Heat map of proteins with significant changes in the RPPA assays of A549, PC-9, H838 and H3122 treated with vehicle or (**a**) 1 μM MK-2206 for 24 hours or (**b**) 10 nM AKT1 siRNA pool for 48 hours. Relative protein levels are color-coded: low (green), median (black), and high (red). Western blot analysis of phospho-MARCKS and other indicated proteins in (**c**) A549 cells and (**d**) PC-9 cells treated with AKT1 siRNA or MK-2206 with/without MARCKS siRNA. (**e**) Migration and invasion assays of A549 cells treated with AKT1 siRNAs or MK-2206 with/without MARCKS siRNAs.

To further explore the potential mechanism underlying LAMC2 upregulation following AKT1 inhibition, we performed RPPA assay to determine the effect of MK-2206 at 1 µM on various signaling pathways in A549, PC-9, H3122 and H838 cells (Fig. 5a; Supplementary Fig. S7a and Table S4). MK-2206 treatment reduced the level of pAKT^S473^ and also resulted in significantly decreased phosphorylation of AKT downstream targets (p4EBP1^S65^, pFOXO1^T24^/pFOXO3a^T32^ and pPRAS40^T246^) in the tested cell lines. Since MK-2206 is a pan-AKT inhibitor, we also performed RPPA assay following AKT1 siRNA knockdown in these cell lines. Knockdown of AKT1 induced many common responses as that of MK-2206 treatment; however not surprisingly, there were also differences among the two treatments (Fig. 5b; Supplementary Fig. S7b and Table S5). For example, AKT1 siRNA decreased the level of p27^kip^ in A549, PC-9 and H838 cells, whereas MK-2206 increased the expression of p27^kip^ in PC-9 and H838 cells (Supplementary Fig. S7a,b). These differences might be due to the inhibitory effect of MK-2206 on AKT2 and AKT3.

In H3122 cells, MK-2206 treatment increased the levels of cleaved-Caspase6^D162^, cleaved-Caspase7^D198^ and cleaved-PARP^D214^ (Supplementary Table S4), and knockdown of AKT1 increased the levels of cleaved-Caspase3^D175^, cleaved-Caspase6^D162^, cleaved-Caspase9^D315^ and cleaved-PARP^D214^ (Supplementary Table S5). These results are consistent with the findings in another EML4-ALK positive cell line H2228 when AKT1 was inhibited (Supplementary Fig. S3a,c). Such changes were not observed in the A549, PC-9 and H838 cells, suggesting that AKT1 provides a crucial survival signaling for EML4-ALK mutant NSCLC cells.

Given that both MK-2206 and AKT1 siRNA enhanced migration and invasion of A549 and PC-9 but suppressed that of H838 cells, we performed RPPA analysis to investigate differences between A549 and PC-9 from H838. Several proteins were increased in MK-2206-treated A549 and PC-9 cells but not in H838 cells, including pMARCKS^S152/156^, AXL and pCrkL^Y207^ (Supplementary Fig. S7a). These molecules have been linked to metastasis in several cancer types^34, 35^. However, of these proteins only pMARCKS^S152/156^ was also elevated in the AKT1 siRNA-treated A549 and PC-9 cells (Supplementary Fig. S7b). The levels of pMARCKS^S152/156^ were 2.8-fold and 1.4-fold higher in MK-2206-treated, and 1.6-fold and 1.3-fold higher in AKT1 siRNA-treated A549 and PC-9 cells, respectively (Supplementary Table S4 and 5). This change was further confirmed in the A549 cells and PC-9 cells by western blot analysis (Fig. 5c,d). However, no significant change of pMARCKS^S152/156^ was observed in the H838 and H3122 cells treated with either MK-2206 or AKT1 siRNA (Supplementary Tables S3 and 4).

Remarkably, we found that siRNA knockdown of MARCKS abolished LAMC2 expression in A549 and PC-9 cells, and the migration and invasion of A549 cells induced by AKT1 siRNA or MK-2206 (Fig. 5c–e). These data suggest that MARCKS is a downstream signaling molecule of AKT1 and its activity is required for the induction of LAMC2 expression and the enhanced migration and invasion resulting from AKT1 inhibition.

Taken together, these results (Figs 4 and 5) suggest that AKT1-MARCKS-LAMC2 forms a feedback regulation loop in the KRAS or EGFR mutant NSCLC cells: AKT1 inhibition induces MARCKS phosphorylation, leading to upregulation of LAMC2 expression to promote cell migration and invasion, whereas increased LAMC2 inhibits AKT1 signaling, thus amplifying the effect of AKT1 inhibition on these cellular processes.

## Discussion

AKT, a key molecule downstream of PI3K, can be inappropriately activated by various oncogenic drivers such as mutant EGFR and KRAS, and is crucial for growth and survival of cancer cells^5, 36^. Targeting AKT has been shown to have anti-tumor activities, and AKT specific inhibitors are currently in clinical development for cancer intervention^14^. AKT regulates diverse biological activities, including cell survival, proliferation, metabolism, angiogenesis, and cell motility^37^. However, conflicting roles of AKT in cell migration, invasion and metastasis have emerged from studies that used different cancer cell lines and model systems. Under certain circumstances, AKT appeared to negatively regulate migration and invasion, and its inhibition promoted metastasis^10, 38^. Thus, understanding the causes of such differences is crucial for future clinical development of AKT inhibitors since chronic administration of these agents might have a promoting effect on the metastatic process.

In this study, we used an *in vivo* mouse metastatic model with repeated intracardiac injections of A549 cells and derivatives to isolate highly metastatic subclones for RPPA and other protein analyses. Our data reveal a negative correlation between AKT1 phosphorylation and the metastatic potential of A549 subclones, implying that AKT1 signaling might, in a way, suppress NSCLC metastasis (Fig. 1 and Supplementary Table S3). Using AKT1 specific siRNAs, we found that inhibition of AKT1 has opposite effects on cell motility and invasion in the NSCLC cell lines with different genetic backgrounds. In KRAS mutant cell lines (A549 and H23) and EGFR mutant cell lines (PC-9 and H1975), AKT1 negatively regulates migration and invasion; whereas in EML4-ALK mutant cell lines (H2228 and H3122) and the EGFR/KRAS wide type cell lines (H838 and H292), it plays a positive role in these cellular processes (Fig. 2). However, inhibition of AKT1 signaling by its siRNAs or MK-2206 in the EML4-ALK mutant cells results in substantial apoptosis. Thus, the manifestation of AKT1 inhibition on migration and invasion of the EML4-ALK mutant cells might also be affected by a strong pro-survival or anti-apoptotic activity of AKT1 in these cells. Our results underscore the crucial influences of genetic backgrounds on the role of AKT1 in different cellular processes.

Since many cellular processes rely on the crosstalk of different pathways^39–41^, AKT1 might form different signaling networks to carry out its biological activities in different cellular contexts. In fibroblasts, AKT1 can phosphorylate Girdin, an actin-binding protein, to promote cell migration^42^. Expression of a constitutively active form of AKT1 in squamous carcinoma cells induces epithelial-mesenchymal transition (EMT) by down-regulation of E-cadherin and up-regulation of vimentin^43^. AKT1 also promotes matrix metalloproteinase-2 (MMP-2) production in mouse mammary epithelial cells and increases MMP-9 via NF-κB activation in fibrosarcoma cells to enhance invasion^44, 45^. However, a paradoxical role of AKT1 has been observed in other cell types^10, 25, 38^. In the MCF-10A mammary epithelial cell line, down-regulation of AKT1 enhances EGF-stimulated migration, and overexpression of AKT1 suppresses EGF-induced cell motility and ERK activation^25^. In breast cancer cell lines, AKT1 suppresses cell migration by inducing degradation of NFAT through E3 ubiquitin ligase HDM2^10^. It has also been shown that AKT1 could inhibit cell migration through suppresseion of the activity of ERK and TSC2^25, 38^. In PC3 prostate cancer cells, down-regulation of AKT1 induces activation of β1-integrins and promotes cell adhesion, migration and invasion^46^. Using a transgenic mouse model, Muller and colleagues found that coexpression of activated AKT1 with oncogenic ErbB2 in mouse mammary epithelial cells enhanced tumorigenesis, but suppressed tumor invasion into the surrounding tissues^47^. In contrast, ErbB2-induced mammary tumors exhibited a higher invasive and metastatic potential in AKT1 knock-out mice^48^. Our data provide evidence to differentiate the conflicting activities of AKT1 in cancer invasion and metastasis based on differences in genetic background.

Given that many AKT inhibitors, including MK-2206, are currently undergoing clincal development, it is of particular importance to determine whether such blockers may have similar effects as that caused by AKT1 siRNA knockdown on NSCLC invasiveness. In our study, MK-2206 enhanced migration and invasion of KRAS or EGFR mutant NSCLC cells. *In vivo*, we found that low dose of MK-2206 (60 mg/kg) enhanced metastasis of A549 cells to brain and bones whereas higher dose (120 mg/kg) had no significant effect (Fig. 3). This is most likely due to different effects of MK-2206 at different dosages on the AKT-mediated cell survival: high dose significantly reduces cell viability thus offsetting the potential effects on the metastatic process, whereas at low dose, its promoting effect on cell invasiveness prevails. It has been shown that combination of MK-2206 with erlotinib in NSCLC cell lines led to synergistic growth inhibition^28^. However, in a phase II clinical trial of advanced NSCLC, MK-2206, in combination with erlotinib, only benefited EGFR wild type NSCLC patients, but not patients with mutant EGFR^49^. Our findings may offer a plausible explanation for the outcome of that trial. Overall, results of phase II trials of MK-2206 have been underwhelming and one of the major issues in the development of inhibitors of this pathway has been patient selection^50^. The results of our study may provide a potential way for negative selection of patients for this treatment.

Exploration of potential mechanism of action has led us to identify MARCKS as a downstream signaling molecule of AKT1 in regulating migration and invasion of KRAS or EGFR mutant NSCLC cells (Fig. 5). MARCKS is the most prominent cellular substrate for protein kinase C (PKC) and binds calmodulin and actin, to regulate actin dynamics^51^. It has been implicated in regulation of cell motility in various types of cells including fibroblasts, myoblasts, and several cancer cell types^52–54^. Knockdown of MARCKS in cancer cells resulted in decreased adhesion, migration and invasion^55^. Although we found that inhibition of AKT1 increased MARCKS phosphorylation, the mechanism of how AKT1 regulates MARCKS signaling remains unclear and warrants further exploration.

Furthermore, we found that inhibition of AKT signaling upregulates LAMC2 protein level, whereas high LAMC2 inhibits AKT signaling. LAMC2 promotes cell motility and high levels of LAMC2 correlate with poorer survival in resected early stage lung adenocarcinoma patients^15^. LAMC2 secreted by cancer cells might also inhibit AKT signaling in the neighboring cells, leading to accumulation of regional LAMC2 proteins to promote invasion and metastasis. Interestingly, we found that LAMC2 upregulation by AKT inhibition is in part mediated by activation of MARCKS. The feedback regulation between AKT1 and LAMC2 apparently exerts an amplifying effect on invasion and metastasis when AKT1 is inhibited.

In conclusion, we found that inhibition of AKT1 signaling promotes migration and invasion *via* MARCKS phosphorylation and LAMC2 upregulation in KRAS or EGFR mutant NSCLC cell lines, but not in EGFR/KRAS wild type cells (Supplementary Fig. S8). These findings underscore the impact of genetic background and cellular context in the regulation of AKT1-mediated invasion and metastasis of NSCLC cells. Our study also provides a strong rationale for clinical development of AKT inhibitors in selected patient groups to avoid the undesired metastatic effect that might result from AKT1 inhibition. Developing MARCKS-targeted therapy may help to improve the therapeutic benefit of AKT inhibitors in NSCLC patients.

## Material and Methods

### Cell culture

Ten NSCLC cell lines purchased from ATCC were used, including two EGFR/KRAS wild type cell lines (H838 and H292), two EGFR mutant cell lines (PC-9 and H1975), two EML4-ALK mutant cell lines (H3122 and H2228) and four KRAS mutant cell lines (A549, H2122, H23 and H358). The genetic characteristics of the NSCLC cell lines were shown in Supplementary Table S1. All cell lines were cultured in RPMI-1640 containing 10% FBS and supplemented with glutamine, penicillin and streptomycin. All cell lines were used at early passages (less than 6 months after resuscitation of the original cells, between Passage 7 to 30) in this paper. All cell lines were tested by using MycoAlertTM Mycoplasma detection kit (Ionza), and proved to be Mycoplasma-free before use. No cell line authentication was done by the authors before initiating this study. LAMC2 transfected cell lines were cultured in the medium described above with the addition of G418, and LAMC2 knockdown cell lines were maintained in Puromycin containing medium, as previously described^15^.

### Antibodies and Reagents

The following antibodies were purchased from Cell Signaling Technology: anti-phospho-AKT (Ser473) (#4060), anti-phospho-AKT (Thr308) (#13038), anti-total AKT (#9272), anti-AKT1 (#2938), anti-AKT2 (#3063), anti-AKT3 (#8081), anti-MARCKS (#5607), anti-phospho-MARCKS (Ser152/156) (#2741), anti-phospho-AKT1 (Ser473) (#9018), anti-phospho-AKT2 (Ser474) (#8599), anti-FOXO1 (#9454), anti-FOXO3a (#12829) and anti-FOXO4 (#9472). Other antibodies used include: anti-phospho-AKT3 (Ser472) (#AP3468a, Abgent), anti-integrin β1 (#610467, BD transduction Laboratories), anti-β-actin (#A5441, Sigma-Aldrich), and anti-LAMC2 (#SC-28330, Santa Cruz). The AKT inhibitor MK-2206 was purchased from Selleckchem.

### RNA interference

Cells were seeded in six-well plates, and transfected with siRNA oligonucleotides using Lipofectamine^TM^ RNAiMAX Reagent (Invitrogen). Forty-eight hours after transfection, cells were collected. Three different AKT1 specific siRNAs (Dharmacon) were used: ACAAGGACGGGCACATTAA (siRNA #1), CAAGGGCACTTTCGGCAAG (siRNA #2), and TCACAGCCCTGAAGTACTC (siRNA #3). AKT2 siRNAs (sc-29197), AKT3 siRNA (sc-38911) and MARCKS siRNAs (sc-35857) were purchased from Santa Cruz.

### Western blotting

Cell lysates were extracted using NP-40 buffer consisting of 150 mmol/L sodium chloride, 1% NP-40, and 50 mmol/L Tris, pH 8.0, supplemented with protease and phosphatase inhibitor (Thermo Scientific). Protein concentrations were measured with Pierce BCA Protein Assay Kit (Thermo Scientific), and 20 μg of total protein were then subjected to electrophoresis in 10% SDS-PAGE and transferred to a PVDF membrane. After blocking in 4% nonfat milk in PBS, the membrane was probed with the indicated primary and secondary antibodies and detected by Western Blot Detection Kit (AbFrontier).

### Cell viability assay

CellTiter-Glo Luminescent Cell Viability Assay (Promega) was used to determine cell viability. For siRNA knockdown experiments, cells transfected with siRNAs for 48 hours were seeded in 96-well plates (5000 cells/well) and incubated for the indicated time. Equal volumes of CellTiter-Glo reagents were then added into each well, and after incubating at room temperature for 10 minutes, the luminescence signals were recorded using Glomax Multi-detection system (Promega). For MK-2206 treatment, cells were seeded in 96-well plates (5000 cells/well) overnight and treated with the indicated concentrations of MK-2206 for 3 days, before measurement of cell viability as described above.

### Reverse phase protein array (RPPA) assay

Cells were seeded in 12-well plates overnight, and then treated with or without 1 μM MK-2206 for 24 hours. Cells lysates were prepared as previously described^56, 57^, printed in triplicate onto nitrocellulose-coated slides, and probed with antibodies recognizing cancer-associated phospho or total proteins. Final signal intensities were acquired and normalized to the total amount of proteins in each individual samples. In A549 R0-R3 test, we probed 114 phospho or total proteins with indicated antibodies, and in the test of A549, PC-9, H3122 and H838 treated with AKT1 siRNA or MK-2206, we screened 169 phospho or total proteins. The antibody lists are shown in Supplementary Table S2. All samples were carried out in triplicate.

### Migration and Invasion assay

For migration assay, cells were counted and plated in triplicates into the top of transwell chambers (Corning) in serum-free medium, while the bottom chambers were supplemented with 10% FBS. For AKT inhibition experiments, 1 μM MK-2206 was added in both chambers. After overnight incubation, cells were fixed and stained with Coomassie blue. The migrated cells were counted from an average of five random visual fields with a microscope. All experiments were carried out in triplicate and repeated three times.

Invasion assays were carried out in the transwell chambers coated with growth factor reduced (GFR)-Matrigel (BD, #356230). Each sample was analyzed in triplicates and three independent experiments were performed.

### Animal experiments

Six-week old athymic nude female mice were purchased from Charles River Laboratories. In the intracardiac injection metastasis model, mice were injected with A549 cells expressing luciferase under anesthesia, as previously described^15^. Briefly, 1cc syringe with a 28 gauge needle (BD, #329410) was loaded with 5 × 10^5^ cells in 100 μL PBS. Then the needle was inserted into the left ventricle of the heart through the second intercostal space, followed by injection of the cells at a very low pace once trace of blood was pumped into the syringe. Following injection, the animal was placed in a clean cage with a heating pad until full recovery. The success of intracardiac injection was further confirmed by *in vivo* bioluminescence imaging immediately after injection (IVIS® Lumina K, PerkinElmer). For MK-2206 experiments, a total of 27 mice were randomly divided into 3 treatment groups: vehicle (30% Captisol), MK-2206 (60 mg/kg), and MK-2206 (120 mg/kg), administered via oral gavage three times per week. Treatments were started on the day after intracardiac injection, and were for two weeks. Mice were monitored by *in vivo* bioluminescence imaging once weekly. All animal experiments were conducted under a protocol approved by the Georgetown University Animal Care Committee.

### Statistics

Unsupervised hierarchical clustering analysis using the Ward’s method was performed in JMP version 5.1 (SAS Institute Inc., SAS, Cary, NC). All other statistical analyses were done using GraphPad Prism 5 software. Data are expressed as the mean ± standard error (SE). Statistical significances were determined by Student’s t-test or ANOVA test with *P* value < 0.05.

## Electronic supplementary material

Supplementary data

## References

[1] Siegel RL, Miller KD, Jemal A (2015). Cancer statistics, 2015. CA: a cancer journal for clinicians.

[2] Herbst RS, Heymach JV, Lippman SM (2008). Lung cancer. The New England journal of medicine.

[3] Group NM-aC (2010). Adjuvant chemotherapy, with or without postoperative radiotherapy, in operable non-small-cell lung cancer: two meta-analyses of individual patient data. Lancet.

[4] Greenman C (2007). Patterns of somatic mutation in human cancer genomes. Nature.

[5] Pao W, Girard N (2011). New driver mutations in non-small-cell lung cancer. The Lancet. Oncology.

[6] Engelman JA (2009). Targeting PI3K signalling in cancer: opportunities, challenges and limitations. Nature reviews. Cancer.

[7] Sos ML (2009). PTEN loss contributes to erlotinib resistance in EGFR-mutant lung cancer by activation of Akt and EGFR. Cancer research.

[8] Engelman JA (2006). Allelic dilution obscures detection of a biologically significant resistance mutation in EGFR-amplified lung cancer. The Journal of clinical investigation.

[9] Yang ZZ (2003). Protein kinase B alpha/Akt1 regulates placental development and fetal growth. The Journal of biological chemistry.

[10] Yoeli-Lerner M (2005). Akt blocks breast cancer cell motility and invasion through the transcription factor NFAT. Molecular cell.

[11] Zhou GL (2006). Opposing roles for Akt1 and Akt2 in Rac/Pak signaling and cell migration. The Journal of biological chemistry.

[12] Cho JH (2015). AKT1 Activation Promotes Development of Melanoma Metastases. Cell Rep.

[13] Ooms LM (2015). The Inositol Polyphosphate 5-Phosphatase PIPP Regulates AKT1-Dependent Breast Cancer Growth and Metastasis. Cancer cell.

[14] Pal SK, Reckamp K, Yu H, Figlin RA (2010). Akt inhibitors in clinical development for the treatment of cancer. Expert opinion on investigational drugs.

[15] Moon, Y. W. *et al*. LAMC2 enhances the metastatic potential of lung adenocarcinoma. *Cell death and differentiation*, doi:10.1038/cdd.2014.228 (2015). 10.1038/cdd.2014.228 PMC4495359 25591736

[16] Liotta LA (2003). Protein microarrays: meeting analytical challenges for clinical applications. Cancer cell.

[17] Nishizuka S (2003). Proteomic profiling of the NCI-60 cancer cell lines using new high-density reverse-phase lysate microarrays. Proceedings of the National Academy of Sciences of the United States of America.

[18] Shieh YS (2005). Expression of axl in lung adenocarcinoma and correlation with tumor progression. Neoplasia.

[19] Koo JS, Kim SH (2011). EGFR and HER-2 status of non-small cell lung cancer brain metastasis and corresponding primary tumor. Neoplasma.

[20] Liu CW (2014). Snail regulates Nanog status during the epithelial-mesenchymal transition via the Smad1/Akt/GSK3beta signaling pathway in non-small-cell lung cancer. Oncotarget.

[21] Chin YR, Toker A (2009). Function of Akt/PKB signaling to cell motility, invasion and the tumor stroma in cancer. Cellular signalling.

[22] Cariaga-Martinez AE (2013). Distinct and specific roles of AKT1 and AKT2 in androgen-sensitive and androgen-independent prostate cancer cells. Cellular signalling.

[23] Chen L (2014). Distinct roles of Akt1 in regulating proliferation, migration and invasion in HepG2 and HCT 116 cells. Oncology reports.

[24] Dillon RL (2009). Akt1 and akt2 play distinct roles in the initiation and metastatic phases of mammary tumor progression. Cancer research.

[25] Irie HY (2005). Distinct roles of Akt1 and Akt2 in regulating cell migration and epithelial-mesenchymal transition. The Journal of cell biology.

[26] Grabinski N (2011). Distinct functional roles of Akt isoforms for proliferation, survival, migration and EGF-mediated signalling in lung cancer derived disseminated tumor cells. Cellular signalling.

[27] Khanna C, Hunter K (2005). Modeling metastasis *in vivo*. Carcinogenesis.

[28] Hirai H (2010). MK-2206, an allosteric Akt inhibitor, enhances antitumor efficacy by standard chemotherapeutic agents or molecular targeted drugs *in vitro* and *in vivo*. Molecular cancer therapeutics.

[29] Salo S (1999). Laminin-5 promotes adhesion and migration of epithelial cells: identification of a migration-related element in the gamma2 chain gene (LAMC2) with activity in transgenic mice. Matrix biology: journal of the International Society for Matrix Biology.

[30] Sarbassov DD, Guertin DA, Ali SM, Sabatini DM (2005). Phosphorylation and regulation of Akt/PKB by the rictor-mTOR complex. Science.

[31] Myatt SS, Lam EW (2007). The emerging roles of forkhead box (Fox) proteins in cancer. Nature reviews. Cancer.

[32] Storz P, Doppler H, Copland JA, Simpson KJ, Toker A (2009). FOXO3a promotes tumor cell invasion through the induction of matrix metalloproteinases. Molecular and cellular biology.

[33] Chandarlapaty S (2011). AKT inhibition relieves feedback suppression of receptor tyrosine kinase expression and activity. Cancer cell.

[34] Li Y (2009). Axl as a potential therapeutic target in cancer: role of Axl in tumor growth, metastasis and angiogenesis. Oncogene.

[35] Rombouts K (2013). Myristoylated Alanine-Rich protein Kinase C Substrate (MARCKS) expression modulates the metastatic phenotype in human and murine colon carcinoma *in vitro* and *in vivo*. Cancer letters.

[36] Manning BD, Cantley LC (2007). AKT/PKB signaling: navigating downstream. Cell.

[37] Altomare DA, Testa JR (2005). Perturbations of the AKT signaling pathway in human cancer. Oncogene.

[38] Liu H (2006). Mechanism of Akt1 inhibition of breast cancer cell invasion reveals a protumorigenic role for TSC2. Proceedings of the National Academy of Sciences of the United States of America.

[39] Fruman DA, Rommel C (2014). PI3K and cancer: lessons, challenges and opportunities. Nature reviews. Drug discovery.

[40] Xue G, Hemmings BA (2013). PKB/Akt-dependent regulation of cell motility. Journal of the National Cancer Institute.

[41] Qiao M, Sheng S, Pardee AB (2008). Metastasis and AKT activation. Cell cycle.

[42] Enomoto A (2005). Akt/PKB regulates actin organization and cell motility via Girdin/APE. Developmental cell.

[43] Grille SJ (2003). The protein kinase Akt induces epithelial mesenchymal transition and promotes enhanced motility and invasiveness of squamous cell carcinoma lines. Cancer research.

[44] Park BK, Zeng X, Glazer RI (2001). Akt1 induces extracellular matrix invasion and matrix metalloproteinase-2 activity in mouse mammary epithelial cells. Cancer research.

[45] Kim D (2001). Akt/PKB promotes cancer cell invasion via increased motility and metalloproteinase production. FASEB journal: official publication of the Federation of American Societies for Experimental Biology.

[46] Virtakoivu R, Pellinen T, Rantala JK, Perala M, Ivaska J (2012). Distinct roles of AKT isoforms in regulating beta1-integrin activity, migration, and invasion in prostate cancer. Molecular biology of the cell.

[47] Hutchinson JN, Jin J, Cardiff RD, Woodgett JR, Muller WJ (2004). Activation of Akt-1 (PKB-alpha) can accelerate ErbB-2-mediated mammary tumorigenesis but suppresses tumor invasion. Cancer research.

[48] Maroulakou IG, Oemler W, Naber SP, Tsichlis PN (2007). Akt1 ablation inhibits, whereas Akt2 ablation accelerates, the development of mammary adenocarcinomas in mouse mammary tumor virus (MMTV)-ErbB2/neu and MMTV-polyoma middle T transgenic mice. Cancer research.

[49] Lara, P. Phase II study of the AKT inhibitor MK-2206 plus erlotinib (E) in patients (pts) with advanced non-small cell lung cancer (NSCLC) who progressed on prior erlotinib: A California Cancer Consortium Phase II trial (NCI 8698). *J Clin Oncol* **32** (2014).

[50] Dienstmann R, Rodon J, Serra V, Tabernero J (2014). Picking the point of inhibition: a comparative review of PI3K/AKT/mTOR pathway inhibitors. Molecular cancer therapeutics.

[51] Hartwig JH (1992). MARCKS is an actin filament crosslinking protein regulated by protein kinase C and calcium-calmodulin. Nature.

[52] Yu, D. *et al*. Myristoylated Alanine-Rich Protein Kinase Substrate (MARCKS) Regulates Small GTPase Rac1 and Cdc42 Activity and Is a Critical Mediator of Vascular Smooth Muscle Cell Migration in Intimal Hyperplasia Formation. *Journal of the American Heart Association* **4**, doi:10.1161/JAHA.115.002255 (2015). 10.1161/JAHA.115.002255 PMC4845127 26450120

[53] Ott LE (2013). Fibroblast Migration Is Regulated by Myristoylated Alanine-Rich C-Kinase Substrate (MARCKS) Protein. PloS one.

[54] Dedieu S, Mazeres G, Poussard S, Brustis JJ, Cottin P (2003). Myoblast migration is prevented by a calpain-dependent accumulation of MARCKS. Biology of the cell/under the auspices of the European Cell Biology Organization.

[55] Micallef J (2009). Epidermal growth factor receptor variant III-induced glioma invasion is mediated through myristoylated alanine-rich protein kinase C substrate overexpression. Cancer research.

[56] Pierobon M, Vanmeter AJ, Moroni N, Galdi F, Petricoin EF (2012). Reverse-phase protein microarrays. Methods in molecular biology.

[57] Pin, E., Federici, G. & Petricoin, E. F. 3rd Preparation and use of reverse protein microarrays. *Curr Protoc Protein Sci* **75**, Unit 27 27, doi:10.1002/0471140864.ps2707s75 (2014). 10.1002/0471140864.ps2707s75 24510676

