# Supplementary material for: Inhibition of AKT1 signaling promotes invasion and metastasis of non-small cell lung cancer cells with K-RAS or EGFR mutations
Source: Sci Rep. 2017 Aug 1;7:7066. doi: 10.1038/s41598-017-06128-9 (PMC5539338; doi:10.1038/s41598-017-06128-9)
Supplement: Supplementary file 1 — Supplementary data [file 41598_2017_6128_MOESM1_ESM.docx]

**Supplementary Material**

**Material and Methods**

**Cell apoptosis assay**

Cells were transfected with siRNAs for 48 hours, and then passaged for another 48 hours incubation. After that, cells were collected and resuspended in 500µl binding buffer for Annexin V-FITC and propidium iodide (PI) staining, and analyzed by flow cytometry (Ex = 488 nm; Em = 350 nm).

**RNA interference**

For knock down experiments of FOXO transcription factors, A549 and PC-9 cells were seeded in six-well plates, and transfected with FOXO siRNA pool oligonucleotides using Lipofectamine^TM^ RNAiMAX Reagent (Invitrogen) for 48 hours, and then cells were collected for western blot. FOXO1, FOXO3a and FOXO4 siRNA pools were purchased from Dharmacon and then mixed together before use. AKT2 siRNAs (sc-29197), AKT3 siRNA (sc-38911) were purchased from Santa Cruz.

**
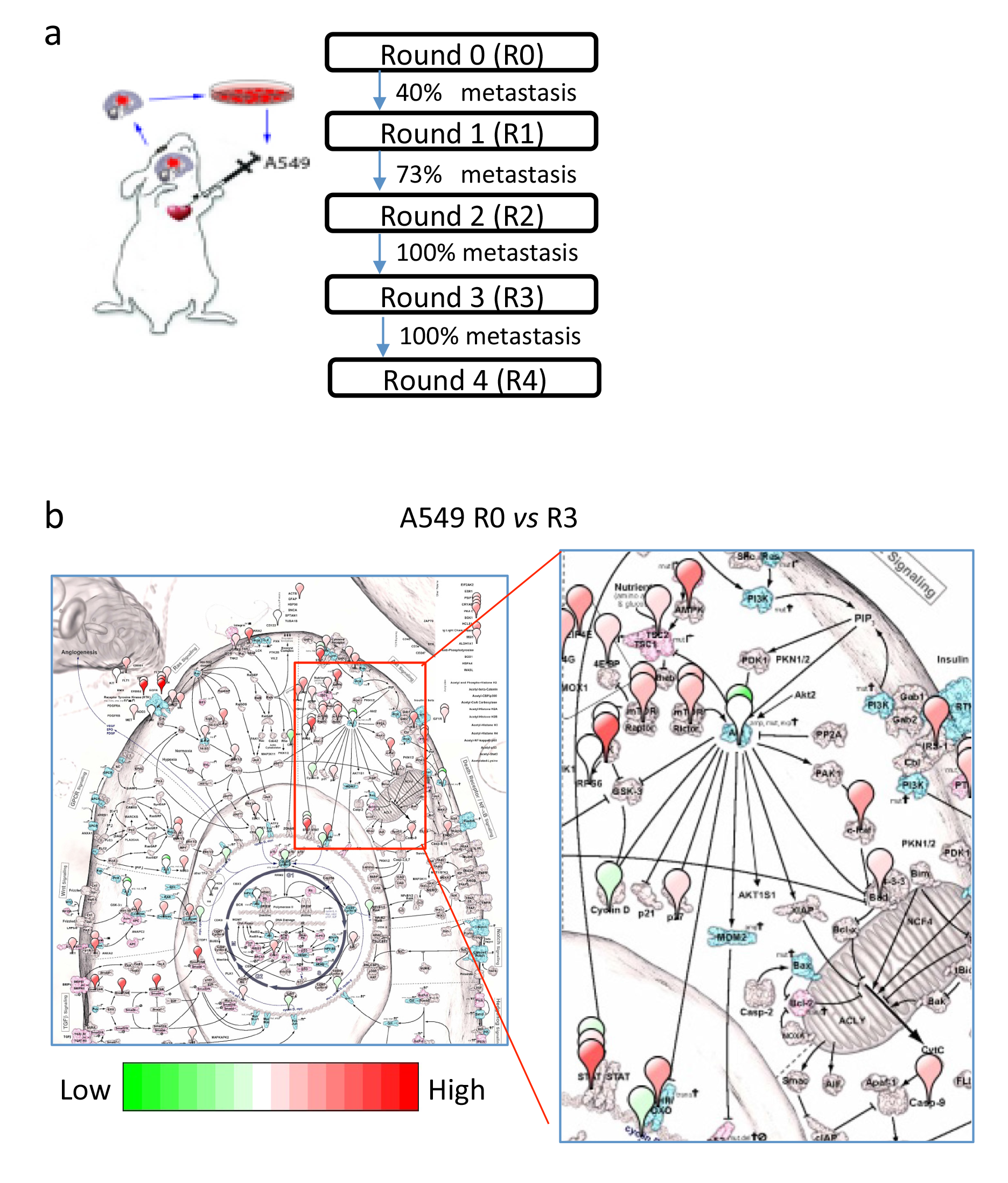
**

**Figure S1. Experimental brain metastasis model and the landscape of RPPA pathway activation.** (A) Schematic representation of the *in vivo* mouse metastasis model. Repeated intracardiac injection of A549 cells were used to select A549 subclones with high metastatic potential to the brain (modified from ([15](#_ENREF_15))). A549-R1, -R2, -R3 and -R4 cells were serially established from brain metastasis by intracardiac injection of original A549 (R0) or derivative cells for stepwise selection. (B) Landscape of protein pathway activations of A549-R3 versus R0. The landscape (left) is plotted by comparing the average fold changes of RPPA data from A549-R3 to that of A549-R0 cells. Each balloon pin is placed over the protein measured. Red indicates pathway activation in the R3 cells, whereas green indicates higher activity of the pathway in the R0 cells. Changes in the PI3K-AKT pathway are highlighted on the right.

**
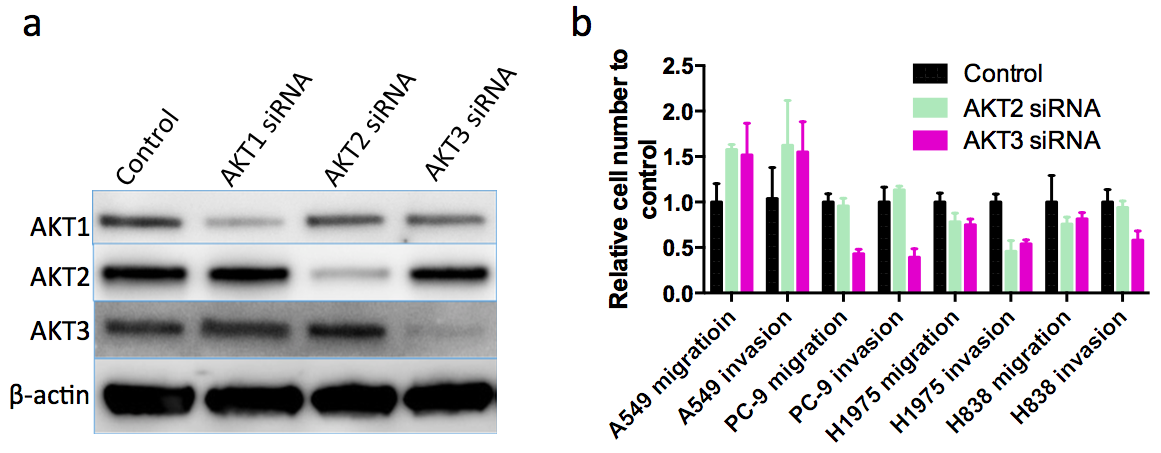
**

**Figure S2. The role of AKT2 and AKT3 in NSCLC cell migration and invasion.**

(A) A549 cells were transfected with the siRNAs pool of AKT1, AKT2 and AKT3 individually for 48 hours, and then cells were collected and the protein levels of AKT isoforms were assessed by western blot. (B) A549, PC-9, H1975 and H838 cells were transfected with AKT2 or AKT3 siRNAs for 48 hours, then, cells were collected for migration and invasion assays.


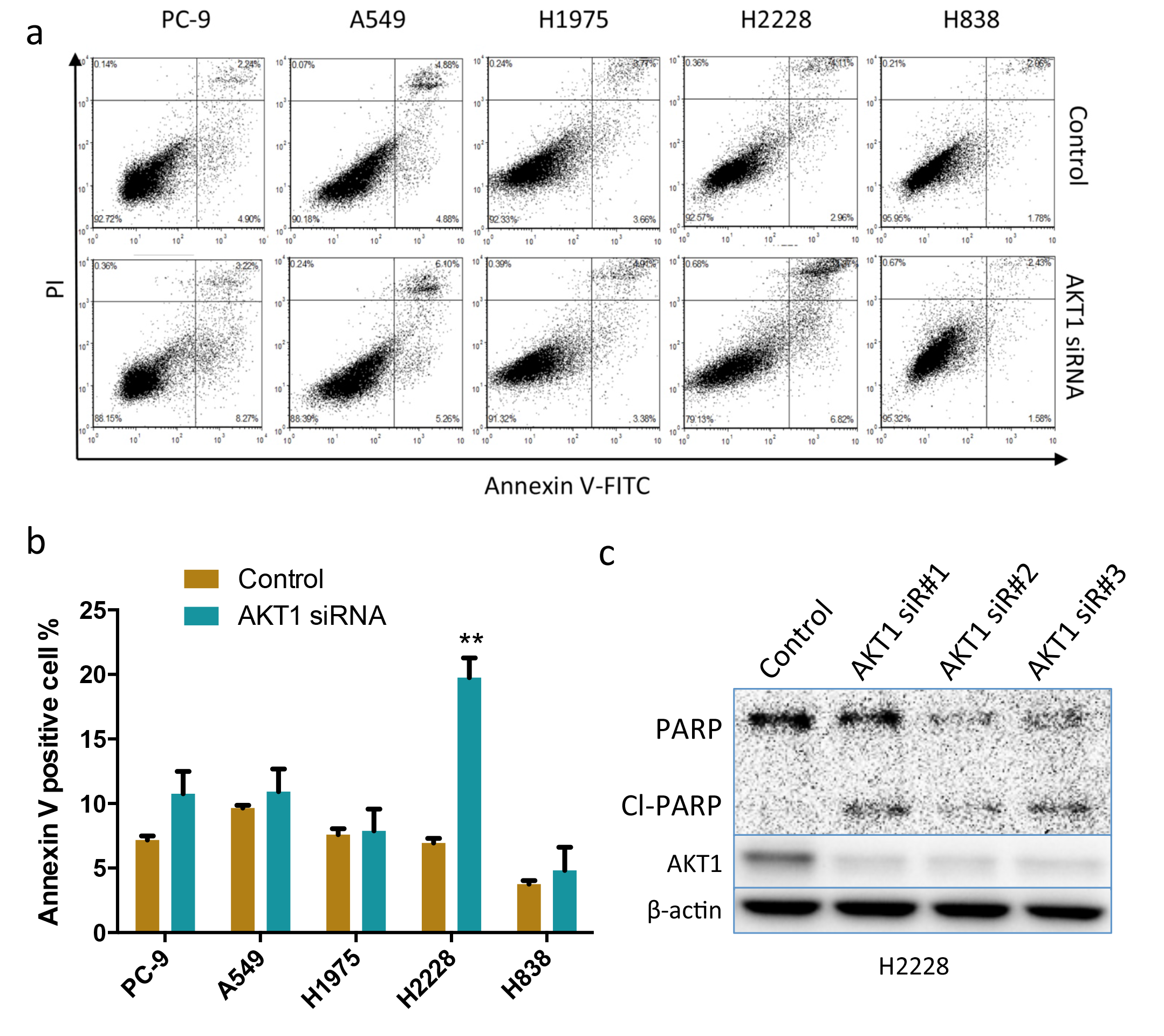


**Figure S3**. **The effect of AKT1 inhibition on NSCLC cell apoptosis.**

(A) Flow cytometric detection of apoptosis by Annexin V staining. A549, PC-9, H1975, H2228 and H838 cells were transfected with AKT1 specific siRNAs for 48 hours, and subjected to flow cytometry after Annexin V and propidium iodide (PI) staining. (B) Quantification of apoptotic cells determined by the Annexin V-Flow cytometric analysis. Values represent means ± SEM of three separate experiments. *: *P* < 0.05, **: *P* < 0.01. (C) Western blot detection of total and cleaved PARP in H2228 cells transfected with three distinct AKT1 siRNA for 48 hours. AKT1 and ß-actin were also probed to monitor AKT1 knockdown and protein loading, respectively.

**
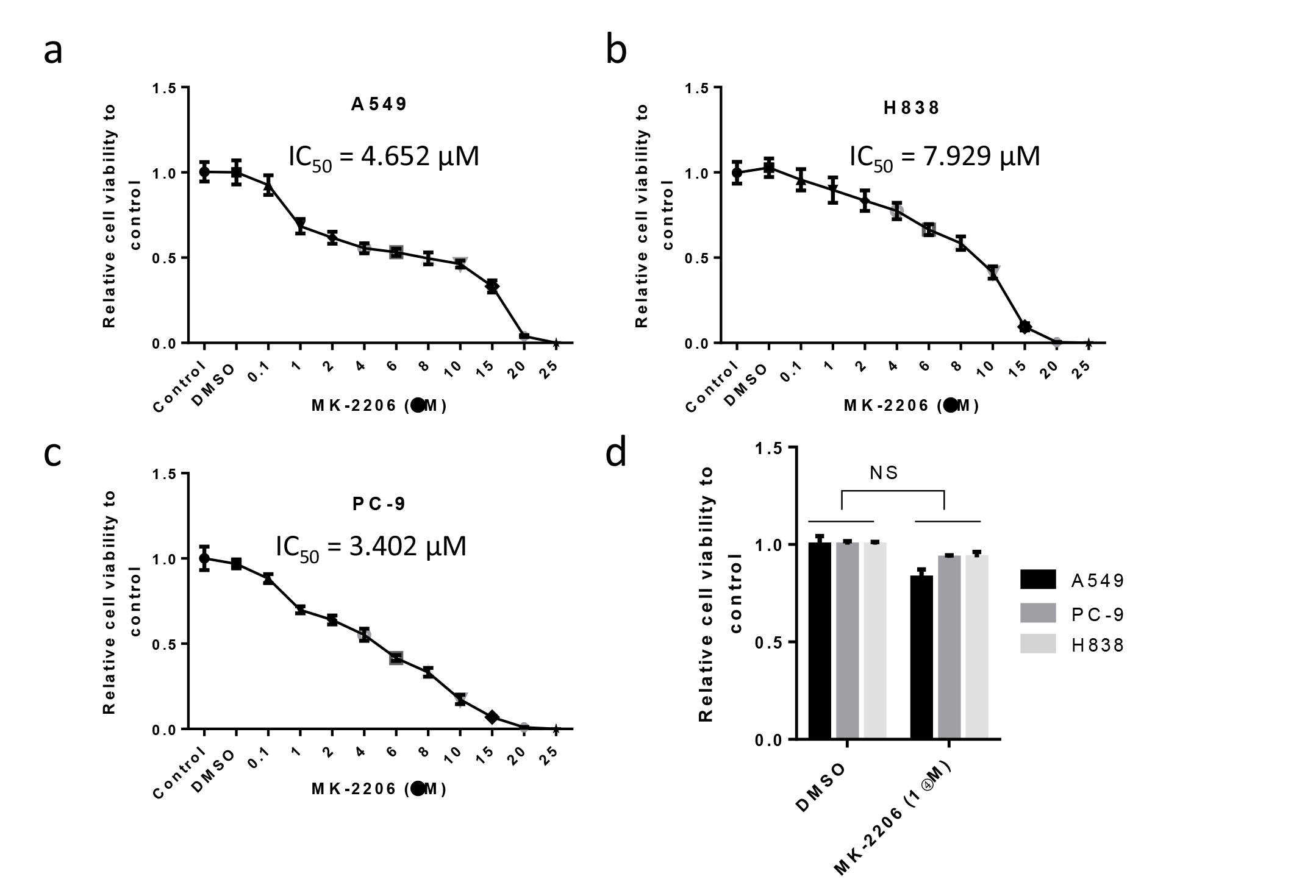
**

**Figure S4. The effect of MK-2206 on the viability of NSCLC cells.** Cell viability of (A) A549, (B) H838 and (C) PC-9 treated with different concentrations of MK-2206 for 72 hours, measured by CellTiter-Glo luminescent cell viability assay. The IC_50_ of MK-2206 was determined using GraphPad Prism. (D) Effect of 1µM MK-2206 on the viability of A549, PC-9 and H838. Cells were seeded into 96-well, and then treated with 1µM MK-2206 for 24 hours before acquiring the data using CellTiter-Glo luminescent cell viability assay. Each bar represents mean ± SE. NS: not significant.


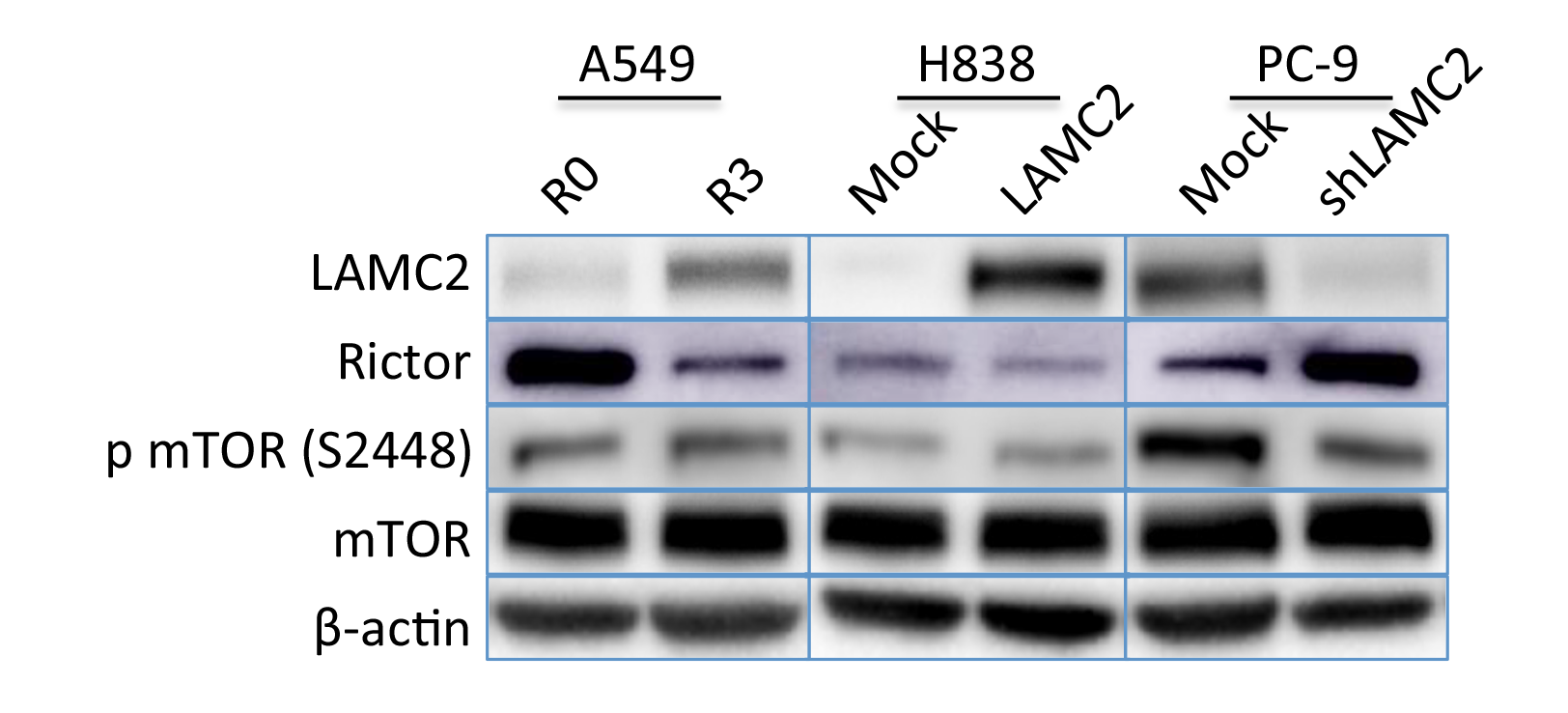


**Figure S5. LAMC2 downregulates Rictor protein levels.** Cells were collected and lysed, then the protein levels of Rictor, phosphorylated-mTOR of S2448, total mTOR and beta-actin were detected by western blot.

**
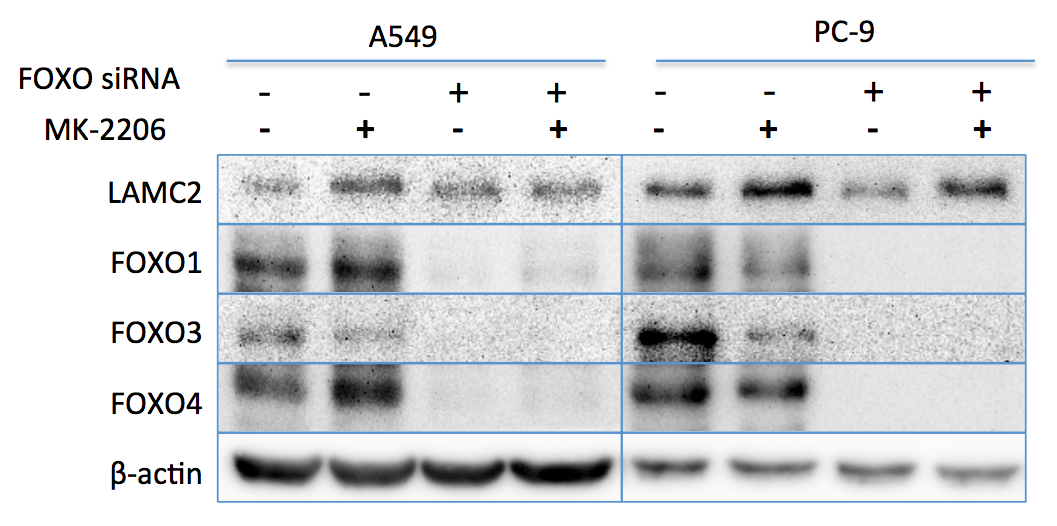
**

**Figure S6. Upregulation of LAMC2 induced by MK-2206 is FOXO-independent.**

A549 and PC-9 cells were transfected with a pool of FOXO1, FOXO3a and FOXO4 siRNAs for 48 hours, followed by treatment with or without 1µM MK-2206 for another 24 hours. The expressions of LAMC2, FOXO1, FOXO3a, and FOXO4 were detected by western blot. Actin was used as a loading control.


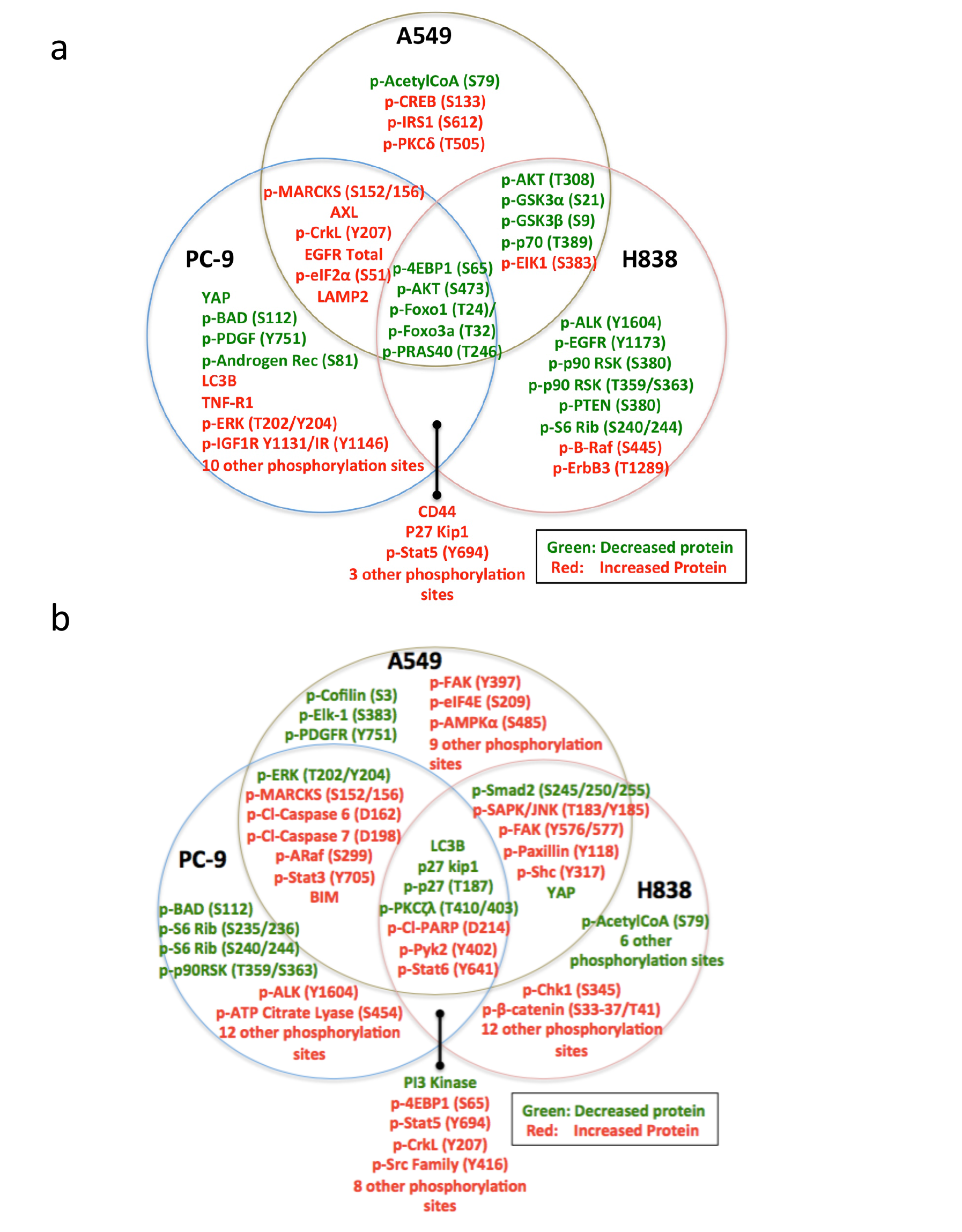


**Figure S7. Highlight of proteins with differential changes in in A549, PC-9 and H838 cells.**

Proteins up- or down-regulated in A549, PC-9 and H838 cells treated with (A) MK-2206 (relative to DMSO control), or (B) AKT1 siRNA (relative to scramble siRNA control), determined by RPPA assay. Highlighted in green: decreased proteins; highlighted in red: increased proteins. Proteins with common changes among different cell lines are listed in the overlapping areas.


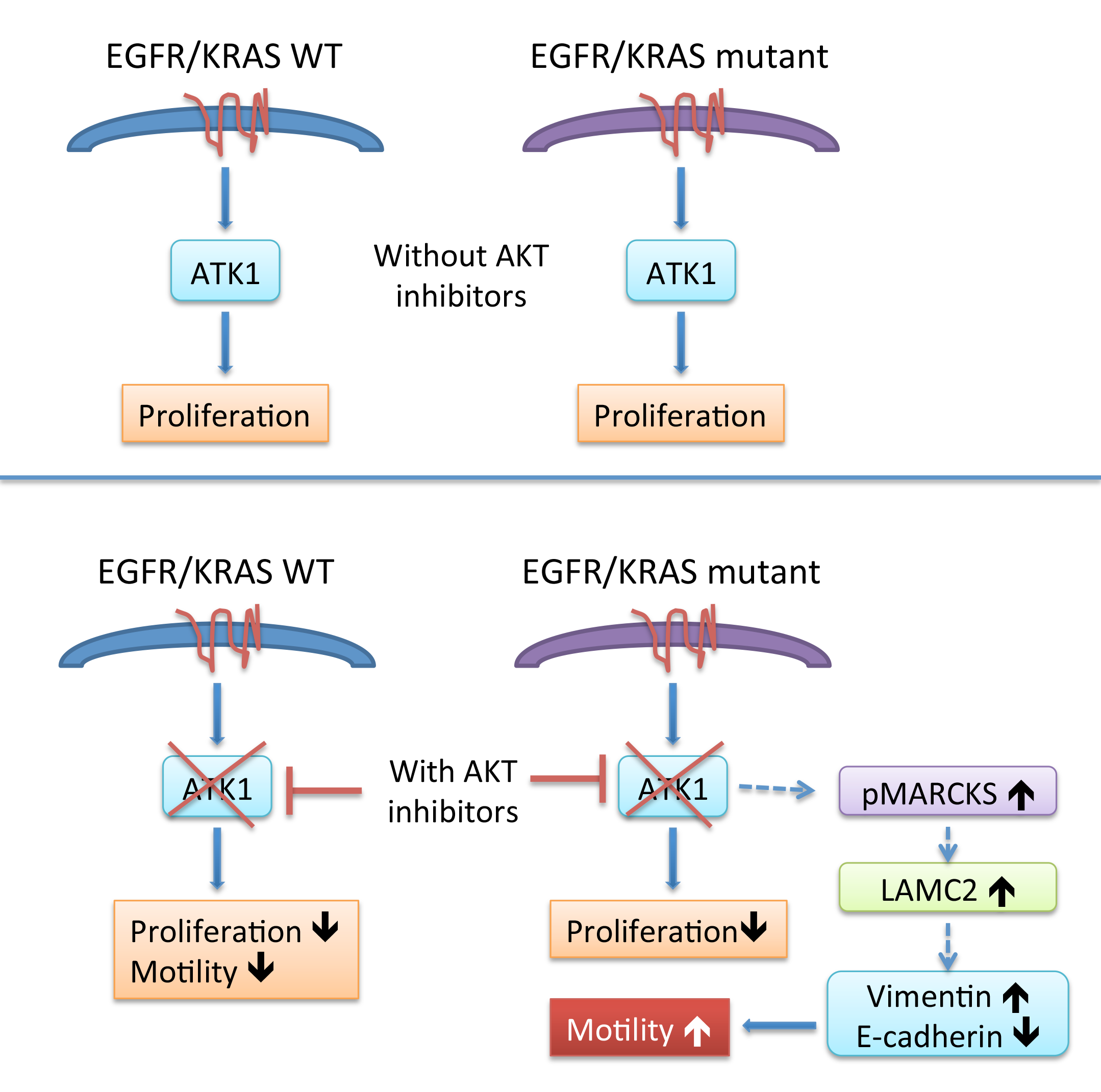


**Figure S8. Graphic representation of AKT1 mechanism of action in the context of EGFR or KRAS mutant cell lines.** Blockade of AKT1 signaling promotes migration and invasion via MARCKS phosphorylation and LAMC2 upregulation in KRAS or EGFR mutant NSCLC cell lines, but not in EGFR/KRAS wild type cells.

**Table S1. The genetic characteristics of the NSCLC cell lines.**

| **NSCLC cell line** | **EGFR mutation** | **KRAS mutation** | **EML4-ALK fusion** |
| --- | --- | --- | --- |
| **H838** | WT | WT | WT |
| **H292** | WT | WT | WT |
| **PC-9** | p.E746_A750delELREA; p.L858R | WT | WT |
| **H1975** | p.T790M; p.L858R | WT | WT |
| **H3122** | WT | WT | EML4-ALK fusion |
| **H2228** | WT | WT | EML4-ALK fusion |
| **A549** | WT | p.G12S | WT |
| **H2122** | WT | p.G12C | WT |
| **H23** | WT | p.G12C | WT |
| **H358** | WT | p.G12C | WT |
| Note: WT = wide type | | | |

**Table S2. The antibody list of RPPA assay.**

| **RPPA antibody list for A549 R0-R3 test** | | | | | | | | | |
| --- | --- | --- | --- | --- | --- | --- | --- | --- | --- |
| 4EBP1S65 | | cABLT735 | | eIF4ES209 | FKHRT24_FKHRL1T32 | | MetY1234-35 | | RetY905 |
| A-RafS299 | | CateninbetaS33-37/T41 | | eIF4GS1108 | GFAP | | mTORS2448 | | RonY1353 |
| Acetyl-CoA Carboxylase S79 | | CD133 | | ElkS383 | GRB2 | | mTORTOTAL | | RosY2274 |
| AKTS473 | | Chk1S345 | | eNOSNOSIIIS116 | GSK3a/bS21/9 | | Musashi | | RSKT356/360 |
| AKTT308 | | cKITY703 | | EphrinA3Y799/A4Y799/A5Y833 | Heregulin | | NFkBp65S536 | | S6 Ribosomal ProteinS240-244 |
| ALDH | | ClCaspase3D175 | | ERaS118 | HistoneH3S10 | | p27T187 | | SAPK_JNKT183/185 |
| ALK | | ClCaspase7D198 | | Erb2/HER2 | IGF1Rbeta | | p38MAPKT180/182 | | SGK1S7 |
| ALKY1586 | | ClCaspase9D330 | | Erb2/HER2Y1248 | IGF1RecY1135_36_InsulinRecY1150_51 | | p53S15 | | ShcY317 |
| AMPKa1S485 | | ClPARPD214 | | ErbB2/HER2Y877 | IGF1RY1131_InsulinRecY1146 | | p70S6KS371 | | Smad1S463/465_Smad5S463/465_Smad8S465/467 |
| AMPKb1S108 | | Cmet | | ErbB3/HER3 | IGFBP7 | | p70S6KT389 | | SmadS245/250/255 |
| ATMS1981 | | CofilinS3 | | ErbB3/HER3Y1289 | Insulin Receptor beta | | p70S6KT412 | | SrcY527 |
| ATRS428 | | CREBS133 | | ErbB4/HER4 | IRS1S612 | | p90RSKS380 | | STAT1Y701 |
| AuroraAT288/BT232/CT198 | | CyclinB1V152 | | ERK1-2T202/Y204 | Jak1Y1022/1023 | | PD/L1 | | STAT3S727 |
| Axl | | CyclinD1 | | ERTOTAL | Jak2Y1007 | | PDGFRbetaY751 | | STAT3Y705 |
| AxlY702 | | E-Cadherin | | FADDS194 | KI67 | | PKACT197 | | STAT5Y694 |
| B-RafS445 | | EGFR | | FAKY576/577 | LC3B | | PLK1T210 | | Survivin |
| BADS136 | | EGFRY1068 | | FGFRY653_654 | LIMK1T508/ LIMK2T505 | | PTEN | | TuberinTSC2Y1571 |
| Beclin1 | | EGFRY1148 | | FKHRL1S253 | M/CSFRecY723 | | PTENS380 | | VEGFRY996 |
| C-RafS338 | | EGFRY1173 | | FKHRS256 | MEK1_2S217_221 | | RbS780 | | YAPS127 |
| **RPPA antibody list for AKT1 siRNA and MK-2206 tests** | | | | | | | | | |
| 4EBP1S65 | Cleaved Caspase 6 D162 | | Est Rec S118 | | LKB1 S428 | PDL1 | | SEK1/MKK4 S80 | |
| AcetylCoA S79 | Cleaved Caspase 7 D198 | | Est Rec Total | | MARCKS S152/156 | PI3 Kinase | | SGK1 S78 | |
| AKT S473 | Cleaved Caspase 9 D315 | | FADD S194 | | MDM2 S166 | PI3K p110gamma | | SGK1 S78 | |
| AKT T308 | Cleaved PARP D214 | | FAK Y397 | | MEK 1/2 S217/221 | PKAC T197 | | Shc Y317 | |
| ALK Y1586 | cMyc | | FAK Y576/577 | | MEK1 S298 | PKC a/BII T638/641 | | SHIP1 Y1020 | |
| ALK Y1604 | Cofilin S3 | | FKHR S256 | | Met Y1234/1235 | PKC delta T505 | | SHP2 Y580 | |
| AMPK alpha1 S485 | cPLA2 S505 | | FKHRL1 S253 | | MHC class I | PKC pan betaII S660 | | Smad1 Ser463/465/ Smad5 Ser463/465/ Smad9 Ser465/467 | |
| AMPK beta S108 | cRaf S338 | | FoxO1 T24/FoxO3a T32 | | MSK1 S360 | PKC S643/676 | | Smad2 S245/250/255 | |
| Androgen Rec S81 | CREB S133 | | Grb2 | | Mst1 T183/Mst2 T180 | PKC theta T538 | | Smad2 S465/467 | |
| ARaf S299 | CrkL Y207 | | GSK 3aB S21/9 | | mTOR S2448 | PKC zeta lambda T410/403 | | SOCS1 | |
| ATG5 g7/2 s31 | CyclinD1 | | GSK3a S21 | | NFkB S536 | PKCa S657 | | SOCS3 | |
| ATP Citrate Lyase S454 | E Cadherin | | GSK3b S9 | | p27 kip1 | PLC gamma1 | | Src Fam Y416 | |
| Aurora A T288/B T232/C T198 | EGFR TOTAL | | Histone H3 S10 | | p27 T187 | PLC gamma1 Y783 | | Stat1 Y701 | |
| Axl | EGFRY 1068 | | Histone H3 S28 | | p38 MAPK T180/Y182 | PP2A A subunit | | Stat2 Y690 | |
| BAD S112 | EGFRY 1148 | | HSP27 S82 | | p53 | PP2A B subunit | | Stat3 S727 | |
| BAD S136 | EGFRY 1173 | | HSP70 s76 | | p53 S15 | PRAS40 T246 | | Stat3 Y705 | |
| Bax | eIF2alpha S51 | | HSP90a T5/7 | | p70S6 S371 | PTEN | | Stat5 Y694 | |
| b-Catenin S33-37/T41 | elF4E S209 | | IGF1R Y1131/IR Y1146 | | p70S6 T389 | PTEN S380 | | Stat6 Y641 | |
| Bcl2 S70 | elF4G S1108 | | IGF1R Y1135-36/IR Y1150-51 | | p70S6 T412 | Pyk2 Y402 | | Survivin | |
| Beclin1 | Elk-1 S383 | | IkBa S32/36 | | p90RSK S380 | Raf S259 | | TNF-R1 | |
| BIM | eNOS/NOS III S16 | | IL6 | | p90RSK T359/S363 | Ras GRF1 S916 | | Tyk2 Y1054/1055 | |
| B-Raf S445 | ErbB2 Y1248 | | IL8 | | PAK1 S199/204 PAK2 S192/197 | Rb S780 | | Ubiquitin | |
| c-Abl T735 | ErbB2/HER2 | | IRS1 S612 | | PAK1 T423/PAK2 T402 | Ret Y905 | | VASP S157 | |
| c-Abl Y245 | ErbB3 T1289 | | JAK1 Y1022/1023 | | PAK2 S20 | Ron Y1353 | | VAV-1 | |
| CD44 | ErbB3 Y1197 | | JAK2 Y1007 | | Paxillin Y118 | S100A7 Calcium binding protein | | VEGFR2 Y1175 | |
| Chk1 S345 | ErbB3/HER3 | | LAMP2 | | PDGFR Y716 | S6 Rib Prot S240/244 | | VEGFR2 Y996 | |
| cKit Y703 | ErbB4/HER4 | | LC3B | | PDGFR Y751 | S6 Rib S235/236 | | Wnt5 a/B | |
| cKit Y709 | ERK T202Y204 | | LIMK1 T508/LIMK2 T505 | | PDK1 S241 | SAPK/JNK T183/Y185 | | YAP | |
| Cleaved Caspase 3 D175 |  | |  | |  |  | |  | |

**Table S3.** **Functional segregation of proteins (with** **ANOVA test p value <0.05) in the RPPA data of A549 R0, R1, R2 and R3 cells.**

| **MITOGENESIS** | **P-value of ANOVA (R0, R1, R2, R3)** | **Pearson's r-value (Related to metastic potential of A549 subclones)** | **Fold Change ( R3 versus R0, highlight fold change < 0.7 or > 1.5)** |
| --- | --- | --- | --- |
| **RTK and LIGANDS** |  |  |  |
| ALK Y1586 | 0.0256 | 0.7995 | 1.07 |
| Axl | 0.0003 | 0.9923*** | 2.45 |
| cABL T735 | 0.0249 | 0.0352 | 0.97 |
| cKIT Y703 | 0.0004 | -0.2518 | 0.94 |
| EGFR | 0.0004 | 0.992*** | 1.64 |
| EGFR Y1068 | <.0001 | 0.9906*** | 2.08 |
| EGFR Y1148 | 0.0007 | 0.9758*** | 1.12 |
| Erb2/HER2 | 0.0014 | 0.1924 | 1.04 |
| Erb2/HER2 Y1248 | <.0001 | 0.9216** | 1.76 |
| FGFR Y653/654 | 0.0284 | -0.3093 | 0.81 |
| Heregulin | <.0001 | 0.851 | 1.41 |
| IGF1R beta | 0.0205 | 0.2477 | 1.12 |
| IGF-1R Y1135/36/IR Y1150/51 | 0.0275 | 0.9577* | 1.12 |
| IGFBP7 | 0.0024 | 0.5291 | 1.22 |
| Met Y1234/35 | 0.0172 | 0.4108 | 1.07 |
| Ret Y905 | 0.0008 | 0.8651 | 1.20 |
| VEGFR Y996 | 0.0014 | 0.8814* | 1.17 |
| **DOWNSTREAM SUBSTRATES** |  |  |  |
| B-Raf S445 | 0.0002 | 0.4818 | 1.11 |
| C-Raf S338 | 0.0056 | 0.5993 | 1.30 |
| Elk S383 | 0.0087 | 0.2287 | 1.02 |
| GRB2 | 0.0245 | 0.6981 | 1.03 |
| IRS-1 S612 | <.0001 | 0.6047 | 1.35 |
| MEK1/2 S217/221 | 0.0116 | 0.9293** | 1.12 |
| p38 MAPK T180/182 | <.0001 | 0.2523 | 1.11 |
| Shc Y317 | 0.0379 | 0.9291* | 1.20 |
| Smad1 S463/465-Smad5 S463/465-Smad9 S465/467 | 0.0004 | 0.9673** | 1.54 |
| Src Y527 | 0.0004 | 0.7347 | 1.39 |
| **CELL SURVIVAL** | **P-value of ANOVA** | **Pearson's r-value** | **Fold Change ( R3 versus R0)** |
| AKT S473 | 0.0004 | -0.9767*** | 0.49 |
| AKT T308 | 0.0364 | -0.3994 | 0.88 |
| 4EBP1 S65 | 0.0148 | 0.0303 | 1.05 |
| AMPKα1 S485 | 0.0004 | 0.828 | 1.44 |
| eIF4E S209 | 0.014 | 0.5969 | 1.32 |
| eIF4G S1108 | 0.0017 | 0.8823* | 1.25 |
| FKHR S256 | <.0001 | 0.7302 | 1.26 |
| mTOR S2448 | 0.0045 | 0.6292 | 1.20 |
| mTOR | <.0001 | 0.3794 | 1.17 |
| p70S6K S371 | 0.0014 | 0.0599 | 0.94 |
| p70S6K T389 | 0.0008 | 0.9431** | 1.55 |
| PKA-C T197 | 0.0032 | 0.5934 | 1.18 |
| PTEN | 0.0232 | 0.9496** | 1.16 |
| PTEN S380 | 0.0053 | 0.9348** | 1.24 |
| S6 Ribosomal Protein S240/244 | 0.0012 | -0.0823 | 0.94 |
| SGK-1 S7 | 0.0205 | 0.8414 | 1.28 |
| TSC2 Y1571 | 0.0046 | 0.9727** | 1.09 |
| **APOPTOSIS and AUTOPHAGY** | **P-value of ANOVA** | **Pearson's r-value** | **Fold Change ( R3 versus R0)** |
| BAD S136 | 0.0047 | 0.7734 | 1.10 |
| Cleaved Caspase 9 D330 | 0.0022 | 0.6363 | 1.22 |
| LC-3B | <.001 | 0.8108 | 1.15 |
| **MOTILITY and CELL ADHESION** | **P-value of ANOVA** | **Pearson's r-value** | **Fold Change ( R3 versus R0)** |
| Catenin beta S33-37/T41 | 0.0104 | 0.7145 | 1.14 |
| Cofilin S3 | 0.0055 | -0.9384** | 0.67 |
| LIMK1 T508/LIMK2 T505 | 0.0018 | 0.2701 | 0.96 |
| **INFLAMMATORY/IMMUNE FUNCTION** | **P-value of ANOVA** | **Pearson's r-value** | **Fold Change ( R3 versus R0)** |
| eNOS/NOSIII S116 | 0.0002 | 0.803 | 1.19 |
| Jak1 Y1022/1023 | 0.0463 | 0.4641 | 1.07 |
| STAT1 Y701 | 0.0172 | 0.7832 | 1.08 |
| STAT3 S727 | 0.0002 | 0.9653** | 1.52 |
| STAT3 Y705 | 0.0223 | -0.6807 | 0.81 |
| Note: RPPA results were analyzed by ANOVA test, and signaling proteins with significant alteration were grouped based on the annotated function of the proteins. The values of correlation coefficient (Pearson’s r-value) were used to measure the relationship between the change of the proteins and the metastatic potential of the cells. The fold changes of proteins (R3 versus R0) are given in the last column, and proteins with fold changes >1.5 or < 0.7 are highlighted in yellow. * represents *P* < 0.05, ** represents *P* < 0.01, *** represents *P* < 0.001 | | | |

**Table S4. Proteins with significant changes in A549, PC-9, H838, H3122 cells after 1μM MK-2206 treatment.**

**Table S5. Proteins with significant changes in A549, PC-9, H838, H3122 cells** **after exposure to AKT1 siRNA.**
